# A High Throughput Biochemical Fluorometric Method for Measuring Lipid Peroxidation in HDL

**DOI:** 10.1371/journal.pone.0111716

**Published:** 2014-11-04

**Authors:** Theodoros Kelesidis, Christian K. Roberts, Diana Huynh, Otoniel Martínez-Maza, Judith S. Currier, Srinivasa T. Reddy, Otto O. Yang

**Affiliations:** 1 Department of Medicine, University of California Los Angeles, Los Angeles, California, United States of America; 2 Exercise and Metabolic Disease Research Laboratory, Translational Sciences Section, School of Nursing, University of California Los Angeles, Los Angeles, California, United States of America; 3 Department of Obstetrics and Gynecology, David Geffen School of Medicine, University of California Los Angeles, Los Angeles, California, United States of America; 4 Department of Molecular and Medical Pharmacology, University of California Los Angeles, Los Angeles, California, United States of America; 5 Department of Microbiology, Immunology, and Molecular Genetics, David Geffen School of Medicine, University of California Los Angeles, Los Angeles, California, United States of America; National Institutes of Health, United States of America

## Abstract

Current cell-based assays for determining the functional properties of high-density lipoproteins (HDL) have limitations. We report here the development of a new, robust fluorometric cell-free biochemical assay that measures HDL lipid peroxidation (HDLox) based on the oxidation of the fluorochrome Amplex Red. HDLox correlated with previously validated cell-based (r = 0.47, p<0.001) and cell-free assays (r = 0.46, p<0.001). HDLox distinguished dysfunctional HDL in established animal models of atherosclerosis and Human Immunodeficiency Virus (HIV) patients. Using an immunoaffinity method for capturing HDL, we demonstrate the utility of this novel assay for measuring HDLox in a high throughput format. Furthermore, HDLox correlated significantly with measures of cardiovascular diseases including carotid intima media thickness (r = 0.35, p<0.01) and subendocardial viability ratio (r = −0.21, p = 0.05) and physiological parameters such as metabolic and anthropometric parameters (p<0.05). In conclusion, we report the development of a new fluorometric method that offers a reproducible and rapid means for determining HDL function/quality that is suitable for high throughput implementation.

## Introduction

There is a continuing search for new biomarkers of atherosclerotic disease. Manipulation of HDL has great potential in reducing cardiovascular risk; however, studies in humans suggest a complex relationship between HDL and atherosclerosis [1], [2]. Lower HDL values are *not* uniformly associated with excess cardiovascular risk while higher HDL levels may *not* always confer a protective benefit [1], [2]. Measuring HDL cholesterol levels provides information about the size of the HDL pool, but does not predict HDL composition or function. Thus, HDL function rather than absolute level may be a more accurate indicator of cardiovascular risk [1], [2].

Due to the complexity of the HDL particles, measurement of HDL function has not been studied extensively in humans [3], [4]. Robust assays to evaluate the function of HDL are needed to supplement the measurement of HDL cholesterol levels in the clinic. Currently, HDL functional properties are most often determined by cell-based assays including the measurement of cholesterol efflux capacity [5]–[8]. However, several limitations render these cell-based assays inaccessible to many researchers and difficult to scale up for large-scale clinical trials and/or routine clinical use [9].

HDL oxidation may contribute to the formation of dysfunctional HDL [10], [11] and we have previously shown that the oxidative properties of HDL are closely associated with HDL function [12]. Along these lines, we previously developed a cell-free assay that measures HDL function by testing the effect of HDL on the production of reactive oxygen species (ROS) after oxidation and conversion of dichlorodihydrofluorescein diacetate (DCFH-DA) to fluorescent DCF (2',7'-dichlorofluorescein) [13]. Although this assay has been used in several studies in humans to study HDL function [3], [4], [14]–[19] and a modified version of this assay has been described [20], [21], it has not found widespread usage due to the oxidative instability of DCFH-DA. We developed an alternative biochemical assay to quantify the HDL redox activity (HRA) that measures the products of redox cycling as the rate of oxidation of the fluorogenic probe dihydrorhodamine-123 (DHR) to fluorescent rhodamine 123 [12]. Despite the relative stability of DHR in contrast to DCF, it was realized that the complexity of the matrix-lipid-probe-ROS interactions in the setting of systemic inflammation might complicate interpretation of the results using the DHR assay [12], [22].

We now describe a new assay to quantify HDL lipid peroxidation (HDLox), a measure of HDL function, in a cell-free biochemical assay. Amplex Red reagent in the absence of cholesterol oxidase and in the presence of horseradish peroxidase (HRP) specifically quantifies the endogenous lipid hydroperoxides of a specific amount of HDL cholesterol. We demonstrate that this approach, in combination with immunoaffinity capture of HDL for HDL isolation, overcomes the confounding factors that affected the readout in the previously described cell free assays of HDL function [12], [22].

## Materials and Methods

### Reagents

Dihydrorhodamine 123 (DHR) was obtained from Molecular Probes (Eugene, OR). DHR was prepared as concentrated stock of 50 mM in dimethyl sulfoxide (DMSO) as previously described [12]. Iron-free HEPES (N-2-hydroxyethylpiperazine-N'-2-ethanesulfonic acid)-buffered saline (HBS, HEPES 20 mM, NaCl 150 mM, pH 7.4) was prepared as previously described [12]. The DHR stock was diluted 1∶1000 in HEPES saline solution to prepare a working solution of 50 µM. Reagents from the Amplex Red Cholesterol Assay Kit (Catalog number A12216, Life Technologies, Grand Island, NY) were used for the new assay. These reagents included the Amplex Red reagent (10-acetyl-3,7-dihydroxyphenoxazine), Hydrogen Peroxide (H_2_O_2_) working solution, Resorufin fluorescence reference standard, horseradish peroxidase (HRP), Cholesterol esterase, reaction buffer (0.5 M potassium phosphate, pH 7.4, 0.25 M NaCl, 25 mM cholic acid, 0.5% Triton X-100). 13(S)-hydroperoxy-9Z, 11E-octadecadienoic acid (13(S)-HPODE) was purchased from Cayman Chemicals (Ann Arbor, MI, USA). Pravastatin sodium (Lot No. M000301, Catalog number P6801) was purchased from LKT Laboratories, Inc.

### HDL and LDL purification and oxidation

HDL and LDL were isolated from cryopreserved human plasma (with or without added sucrose) by ultracentrifugation, fast performance liquid chromatography (FPLC), or precipitation with polyethylene glycol as previously described [12], [23]–[26]. HDL was also oxidized *in vitro* with 13(S)-HPODE as described [16]: human HDL (5 µg HDL-cholesterol/ml) was incubated with 13(S)-HPODE (0.5 µg/ml), for 60 min (co-incubation).

### Immunoaffinity capture of HDL

We have previously used immunoaffinity capture of HDL to study changes in the proteome associated with dysfunctional HDL [14], [18], [19]. Briefly, 96-well polyvinyl chloride microfilter plates (BD Biosciences) were precoated with 1–5 µg/ml of chicken anti-human HDL antibodies (GenWay Biotech, San Diego, CA) overnight at 4°C. Following incubation of the pre-coated plates with individual plasma samples diluted at 1∶10 with 1X PBS, serum at 1∶20 dilution, or HDL at 1∶2 dilution, the plates were washed thoroughly. Detection of HDL was confirmed by HDL-capturing sandwich enzyme-linked immunosorbent assays (ELISA) as described previously using corresponding primary antibodies to human ApoA-I at 1∶2,500 dilution [14], [18], [19]. In addition, total HDL was captured using polyclonal antibodies included in 5 commercially available kits [Genway Inc (kit A); Biotang Inc (kit B), Cusabio Inc (kit C), China; WKEA Inc, China (kit D); Wuhan EIAab., Ltd; China (kit E)) according to the manufacturers' instructions.

### Measurement of HDL cholesterol

HDL cholesterol was quantified using a standard colorimetric assay (Thermo DMA Co., San Jose, CA, USA) as previously described [12]. Samples were also assayed by the UCLA Clinical Laboratory for total cholesterol, high-density lipoprotein (HDL), non-HDL cholesterol and triglycerides (TG) using the Olympus AU400 Chemistry Analyzer.

### Measurement of total HDL protein

The total HDL protein “captured” in each well was measured using the BCA Protein Assay as previously described [14], [17]–[19]. In addition, the total HDL protein concentration for each sample was measured using total HDL ELISA and the above commercially available kits (Kits A, B) according to the manufacturer's instructions.

### Detection of albumin

50 µl of plasma (n = 10) was added into 96 wells and was isolated using immunoaffinity capture of HDL according to the manufacturer's instructions (Kit A). After 5 washes, 300 µl of albumin bromocresol green reaction (BCG) reagent (Thermo Scientific Inc) were added to each well and after 90 second incubation at 37°C optical density was measured at 630 nm. Results are expressed as % relative value compared to the positive control (50 µl of plasma). Detection of albumin was also confirmed by albumin ELISA according to the manufacturer's instructions (Pierce) using corresponding antibodies to human albumin conjugated to HRP.

### HDL inflammatory index

The HDL-inflammatory index (HII) was determined for each subject's HDL as described previously [8], [13]. Values in the absence of HDL were normalized to 1.0. The values obtained in the presence of the test HDL were divided by the value obtained in the absence of HDL to yield the HII. Values >1.0 after the addition of the test HDL indicated proinflammatory HDL; values <1.0 indicated anti-inflammatory HDL.

### DHR-based cell-free assay of HDL function

The DHR assay was performed as previously described [12]. Briefly, quadruplicates of HDL (5 µg of cholesterol unless otherwise specified were added to 96-well plates (polypropylene, flat bottom, black, Fisher Scientific, USA). HBS was added to each well to a final volume of 150 µl, followed by addition of 25 µl of the working solution of 50 µM DHR, for a total volume of 175 µl. Immediately following DHR addition, the plate was protected from light, placed in the fluorescence plate reader and fluorescence of each well was assessed at two minute intervals for an hour with a Synergy 2 Multi-Mode Microplate Reader (Biotek, Vermont, USA), using a 485/538 nm excitation/emission filter pair with the photomultiplier sensitivity set at medium. The oxidation rate was calculated for each well as the slope for the linear regression of fluorescence intensity over 60 minutes, expressed as FU minute-1 (fluorescence units per minute). HDLox was calculated as the mean of quadruplicates for the wells containing the HDL sample.

### Amplex red assay

#### Method A: Use of PEG precipitation for HDL isolation

ApoB depleted serum was isolated from human plasma using PEG precipitation and then a specific amount of HDL cholesterol (5 µg; quantified using a standard colorimetric assay, Thermo, CA, USA) was added to 96-well plates (polypropylene, flat bottom, black, Fisher Scientific, USA) in quadruplicates with the appropriate volume of 1X reaction buffer for a total volume of 50 ul per reaction well. A 2 mM resorufin solution was used to prepare a standard curve to determine the moles of product produced in the Amplex Red reaction. The appropriate amount of 2 mM resorufin reference standard was diluted into 1X reaction buffer to produce resorufin concentrations of 0 to ∼20 µM). 1X reaction buffer without cholesterol was used as a negative control. A 20 mM H_2_O_2_ working solution was used as a positive control. A volume of 50 µL was used for each reaction. 50 µl of HRP/catalase solution containing 1–10 U/mL HRP and 1–4 U/ml catalase was then added followed by an incubation for 60 minutes. 50 µl of 300 µM of Amplex Red reagent (Invitrogen) containing 0.2 U/mL cholesterol esterase were then added to each microplate well containing the samples and controls. The fluorescence of each well was assessed at one-minute intervals over 60 minutes with a plate reader (Biotek, Vermont, USA), using a 530/590 nm filter pair. The fluorescence at each timepoint can be expressed as: 1) relative to the maximum fluorescence observed over 60 minutes 2) relative to fluorescence of the corresponding timepoint of a reference control sample 3) as specific concentration of resorufin (µM) extrapolated from the resorufin standard curve at the specific timepoint using 4 parameter logistic regression analysis. The slope of the reaction of the Amplex Red reagent with the endogenous hydroperoxides present in HDL in the absence of cholesterol oxidase, corresponds to the endogenous HDLox of each sample and was calculated over 60 min using the Gen5 2.01 software (Biotek, Vermont, USA). Alternatively, a specific volume (50 µl) of apoB-depleted serum was added in each well in quadruplicates and the mean fluorescence readout (slope) was normalized by the HDL cholesterol concentration of each sample as measured by the clinical lab (mg/dL). A buffer blank was measured in each assay, and the fluorescence readout in the buffer blank was subtracted from each sample. The plate was protected from light during the assay to minimize artifactual oxidation by light. A step by step description of the assay is provided in Table S1 and Figure S1.

#### Method B: Use of HDL immunocapture for HDL isolation

The following matrices were used for HDL capture in 96 well plates: 1) specific amount of commercially available purified HDL (Sigma) (5 µg of HDL cholesterol) 2) specific amount of purified HDL isolated by ultracentrifugation (5 µg of HDL cholesterol) 3) specific amount of apo-B depleted serum isolated by PEG precipitation (5 µg of HDL cholesterol) 4) specific volume of plasma or serum (100 µl) 5) specific volume of apo-B depleted serum (100 µl). These matrices were then added into 96 wells and HDL was isolated using immunoaffinity capture of HDL as described in Methods. The Amplex Red assay was performed as described above. When a specific volume (100 µl) of apoB-depleted serum or plasma/serum was added in each well in quadruplicates, the mean fluorescence readout (slope) was normalized by the HDL cholesterol concentration of each sample as measured by the clinical lab (mg/dL).

### Human subjects

#### Subjects with coronary artery disease (CAD)

Blood samples were collected from patients with coronary artery disease (CAD) or equivalent as defined by the National Cholesterol Education Program Adult Treatment Panel III criteria [27] and were collected from patients referred to the cardiac catheterization laboratory at the Center for Health Sciences at the University of California, Los Angeles. After signing a consent form approved by the Human Research Subject Protection Committee of the University of California, Los Angeles, the patient donated a fasting blood sample collected in a heparinized tube. Plasma samples were also randomly selected from pre-treatment samples remaining from a previously described study in which all patients had coronary artery disease or equivalent [8]. All of these patients were on a statin [8].

#### Human Immunodeficiency Virus (HIV-1)-infected individuals

Fifty HIV-1-infected individuals on combination antiretroviral therapy (ART) with suppressed viremia (below 50 copies of RNA/ml) (48 males and 2 females; median age 44, range 18–53 years) were recruited at the University of California, Los Angeles (UCLA) as previously described [8]. These patients had no documented coronary atherosclerosis and normal total cholesterol (200 mg/dl), LDL cholesterol (130 mg/dl), HDL cholesterol (males, >45 mg/dl; females, >50 mg/dl), and triglycerides (<150 mg/dl), were not receiving hypolipidemic medications and were not diabetic. Fifty normal volunteers (42 males and 8 females; median age 42, range 18–57 years), matched by age and gender, were recruited under a protocol approved by the Human Research Subject Protection Committee of the University of California, Los Angeles (UCLA IRB). Blood samples from a previously completed matched cohort study that was designed to investigate the role of ART therapy and HIV-1 infection on the risk for subclinical atherosclerosis and its progression were also used for validation of the new assay; the study design has been previously published [28]. In this cohort subjects were enrolled as risk factor (age, sex, race/ethnicity, smoking status, blood pressure, and menopause status)-matched triads of HIV-1-infected individuals with viremia <500 RNA copies/ml with (n = 29) or without (n = 26) use of protease inhibitor (PI) therapy, and HIV-1-uninfected subjects (n = 36).

#### Healthy subjects

Blood bank specimens were collected from healthy young blood donors according to previously well-defined criteria [29]–[31]. More specifically the donors were young (range 19–40 years old) had no known underlying diseases including diabetes, were known to have normal lipid profile and were not receiving hypolipidemic medications.

#### Exercise study participants

In this cross sectional study, 90 young adult males, ages of 18–30 were recruited and categorized into 3 phenotypes based on training status and Body Mass Index (BMI): lean trained (LT, n = 30, ≥4 d/week resistance training (RT), BMI<25 kg/m2), overweight trained (OT, n = 30, ≥4 d/week RT, BMI>27 kg/m2) and overweight untrained populations (OU, n = 30, no structured exercise regimen, BMI>27 kg/m2). Overweight untrained subjects participated in only light intensity physical activity ≤2 times/wk. Participants with overt chronic disease symptoms, as indicated by screening, comprehensive history and/or physical examination, were excluded from the study. Potential participants who had documented cardiovascular disease or used tobacco products or medications that influence cardiovascular function, body composition or insulin indices in the prior 6 months were excluded from the study. All of the study protocols were approved by the UCLA IRB.

### Mice

ApoE and LDLR null mice originally purchased from the Jackson Laboratories on a C57BL/6J background were obtained from the breeding colony of the Department of Laboratory and Animal Medicine at the David Geffen School of Medicine at UCLA as previously described [32]. The mice were maintained on a Western diet (Teklad, Harlan, catalog # TD88137) for 2 weeks and a group of mice was also treated with pravastatin at 12 µg/ml drinking water, or approximately 50 µg per day for two weeks. All experiments were performed using protocols approved by the Animal Research Committee at UCLA.

### Data collection

Metabolic syndrome was defined by National Cholesterol Education Program (NCEP) criteria [27]. In the HIV cohort fasting glucose, insulin, lipids, high-sensitivity C-reactive protein (hs-CRP), cardiovascular disease-related measurements, CD4+ T cell counts and HIV-1 RNA levels were previously determined [28]. Carotid artery intima-media thickness (CIMT) of the far wall of the right common carotid artery was measured at baseline and longitudinally as previously published [28]. In the exercise study measurements of serum glucose, serum insulin, homeostasis model assessment for insulin resistance (HOMA), oxLDL, hs-CRP, body composition parameters, anthropometric parameters, blood pressure (i.e. brachial systolic and diastolic pressure, bSBP, bDBP) maximal strength testing, subendocardial viability ratio (SEVR) were performed as previously described [33].

### Statistical analysis

Statistical analyses were performed with the use of Stata statistical software 12 (StataCorp LP., College Station, TX). Group means were compared using the Student's t-test for unpaired variates with p<0.05 considered to be statistically significant. Correlation coefficients between variables were calculated using least squares linear regression. In the HIV cohort, conditional logistic regression modeling for matched pairs data stratified by triad evaluated associations with CIMT progression. Covariates significant in the univariate analysis (p<0.05) were examined together in multivariate analysis. In the exercise study, post-hoc Pearson correlation analyses were used to determine the relationships between HDLox and cardiovascular and metabolic disease risk biomarkers.

## Results

### Amplex Red can specifically measure lipid peroxidation of a specific amount of HDL

**Figure 1:**
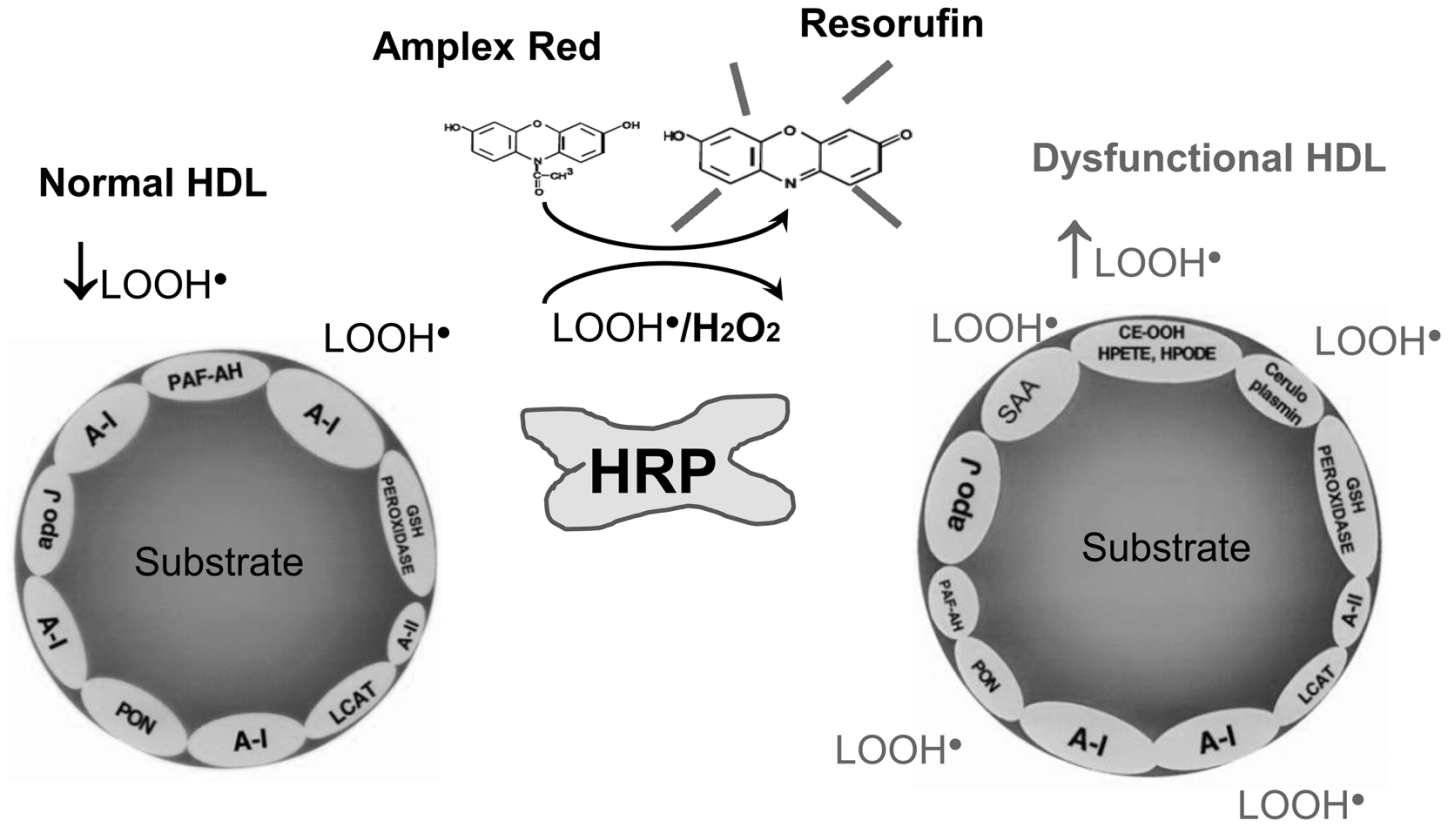
Principle of the Amplex Red assay of HDL function. 1. The acute-phase (AP) reaction favors the formation of dysfunctional HDL. In the basal state, HDL contains apoA-I and apoJ as well as 4 enzymes, paraoxonase (PON) and platelet-activating factor acetylhydrolase (PAF-AH), lecithin: cholesterol acyltransferase (LCAT), and plasma reduced glutathione selenoperoxidase (GSH peroxidase) that can prevent the formation of or inactivate the LDL-derived oxidized phospholipids found in oxidized LDL. As a result, in the basal state, HDL may be considered anti-oxidant. As previously published (Navab M et al. Arterioscler Thromb Vasc Biol 2001; 21: 481–488), during the acute-phase reaction, A-I may be displaced by the pro-oxidant acute-phase reactant Serum amyloid A (SAA). Another pro-oxidant acute-phase reactant, ceruloplasmin, associates with HDL as does the anti-oxidant acute phase reactant apoJ. PON, PAF-AH, and LCAT decrease in HDL during the acute-phase reaction, and the lipid hydroperoxides (LOOH) 5-hydroperoxyeicosatetraenoic acid (HPETE), hydroperoxyoctadecadienoic acid (HPODE), and cholesteryl linoleate hydroperoxide (CE-OOH) increase in HDL. The net effect of the changes in HDL during the acute-phase reaction is the production of pro-oxidant, HDL particles (AP-HDL or dysfunctional HDL). 3. HDL can be isolated using different methods such as ultracentrifugation, PEG precipitation and immunoaffinity capture. Using immunoaffinity capture of HDL and commercially available antibodies against total human HDL, HDL is isolated from a specific volume (e.g. 100 ul) of either a) non EDTA plasma b) serum or c) apoB depleted serum 4. Amplex Red (N-acetyl-3, 7-dihydroxyphenoxazine) reagent is a colorless substrate that reacts with hydrogen peroxide (H_2_O_2_) in the presence of horseradish peroxidase (HRP) with a 1∶1 stoichiometry to produce highly fluorescent resorufin (excitation/emission maxima  = 570/585 nm). This highly stable, sensitive and specific fluorogenic substrate for HRP has been widely used to develop a variety of fluorogenic assays for enzymes that produce hydrogen peroxide. For example Amplex Red reagent coupled with the enzymes cholesterol oxidase and HRP permit the ultrasensitive quantitation of HDL cholesterol based on lipid peroxidation. The biochemistry of lipid peroxidation has been well established (Free Radical Research 2010∶44, 1098–1124) and during this reaction there is formation of reactive oxygen species (ROS) such as peroxyl and alkoxyl radicals (ROO-, RO-; LOOH-) that have previously been shown to react with the Amplex Red reagent to form fluorescent resorufin ((J. Biol. Chem. 284, 46–55; J Biol Chem. 2010 May 28; 285(22): 16599–605). Resorufin is produced by the reaction of the Amplex Red reagent with H_2_O_2_ produced from the cholesterol oxidase-catalyzed oxidation of cholesterol. In the absence of cholesterol oxidase, the “endogenous” hydroperoxide content of a specific amount of HDL cholesterol can be quantified in the presence of HRP and Amplex Red. High hydroperoxide content of a specific amount of HDL cholesterol has previously been shown to be significantly associated with abnormal HDL function. The background production of hydroxyradicals as a result of air oxidation of the buffer (based on the readout of the blank well that contains Amplex Red reagent and buffer) is subtracted from the fluorescent readout of each well. In addition, to better evaluate the LOOH-dependent oxidation, catalase (not shown in figure) may be added in the medium to remove rapidly the formed H2O2 so that the detected increase in fluorescence may be attributed mainly to lipid LOOH release.

Amplex Red in the presence of the enzyme cholesterol oxidase has been reliably used to quantify cholesterol content of HDL based on lipid peroxidation of HDL [34], [35]. The biochemical reaction of the Amplex Red Reagent in the presence of HRP and H2O2 to produce highly fluorescent resorufin and measure peroxides is well established [34], [35]. Horseradish peroxidase is known to catalyze both the H2O2- and LOOH-dependent oxidation of non-fluorescent Amplex Red to fluorescent resorufin red [36], [37]. Thus, to evaluate the LOOH-dependent oxidation, catalase may be added in the incubation medium to remove rapidly the formed H2O2 so that the detected increase in fluorescence may be attributed to lipid LOOH release [36], [37]. Using a modification of a previously published method that was used to measure lipid hydroperoxide content [36], [37], we detected HDL LOOH release using Amplex Red, horseradish peroxidase, and catalase. In the absence of cholesterol oxidase and in the presence of HRP and catalase, Amplex Red specifically quantifies the endogenous lipid hydroperoxides of a specific amount of HDL cholesterol (Fig. 1). Using this modification (no cholesterol oxidase), for the same amount of HDL cholesterol differences in the rate of lipid peroxidation between different samples (as detected by the Amplex Red reagent) would correspond to differences in HDL lipid peroxidation (Fig. 1).

### Lipid probe interactions are still present when using Amplex Red in fluorescent assays of HDL function

Since use of fluorescent assays of HDL function may allow study of the role of the functional phenotype of HDL in the setting of large scale studies [12] especially with the use of a robust, reproducible, high throughput method to extract HDL from patient serum such as PEG precipitation [4], we determined the (apo) B-depleted serum-Amplex Red interactions in patients with inflammatory conditions such as HIV versus healthy subjects. Without HDL, Amplex Red exposed to air became oxidized (and therefore fluorescent) at a linear rate within 60 minutes. The rate of oxidation of Amplex Red was significantly less with added HDL, and different HDL samples (from patient with dysfunctional HDL compared to healthy subjects) showed clearly different effects in this regard (Fig. S2). Thus, apoB depleted serum-probe interactions were also present with Amplex Red similarly to the previously used fluorescent probes [22].

### Use of Amplex Red in combination with HRP overcomes lipid probe interactions when measuring lipid peroxidation of HDL

To determine whether the reduction in the fluorescence signal of Amplex Red after addition of HDL can be minimized or abolished by adding an enzyme that specifically catalyzes the lipid peroxidation and the oxidation of Amplex red [38]–[40], we tested the effect of addition of different concentrations of HRP on the oxidation rate of Amplex Red in the presence of a specific amount of HDL cholesterol. Determination of the rate of production of resorufin was performed by measuring the slope of fluorescence increase during the first 60 minutes, when the rate of oxidation is linear (Fig. S3). Increasing concentrations of HRP 0.5–4 U/ml increased the oxidation rate of Amplex Red in the setting of lipid peroxidation of specific amount of HDL, compared to when Amplex Red was used without any HRP (Fig. S2). These differences could be demonstrated using both purified HDL isolated by ultracentrifugation and FPLC and apoB depleted serum (Fig. S2), a matrix that is characterized by more prominent lipid probe interactions [22]. Addition of catalase (1–4 U/ml) did not significantly change the relative differences in HDLox between different groups of samples (dysfunctional HDL versus normal HDL). Thus, HDLox can be quantified with the use of Amplex Red and HRP with minimal lipid-probe interactions.

### Amplex Red can reliably measure lipid peroxidation of a specific amount of HDL (HDLox)

**Figure 2:**
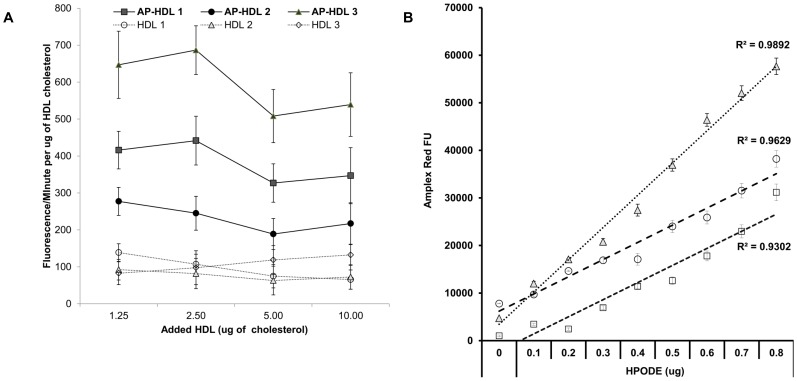
Assay performance. **A**. Linearity of the Amplex Red Assay of HDL lipid peroxidation when ≤10 ug of HDL cholesterol is added. HDL was isolated by ultracentrifugation from 3 HIV infected patients known to have acute phase HDL (AP-HDL) and 3 patients with normal HDL (as determined using a previous assay of HDL function; J Lipid Res. 2011; 52: 2341–51). HDL was then added in varying concentrations (cholesterol) to 300 µM Amplex Red in a 96 well flat bottom plate and the rate of change in fluorescence was measured as in Figure S2 in the presence of 4 U/ml of HRP. The rates of change in fluorescence were normalized against the added HDL cholesterol amount (in µg of cholesterol as determined by a cholesterol assay) and are plotted (means and standard deviations) against the amounts of added HDL. Similar results were observed when HDL cholesterol isolated by PEG precipitation was added to the reaction. B. The linearity of the assay was also demonstrated using *in vitro* oxidation of HDL by adding increasing amounts of pre-formed lipid peroxides. *In vivo* studies have shown that 13(S)-H(P)ODE is an *in vivo* generated lipid oxidant that has a key role in atherogenesis and contributes to formation of dysfunctional HDL (Drug Metab Lett. 2010; 4: 139–48). HDL from 3 different healthy subjects (1 µg) was oxidized *in vitro* with increasing amounts of HPODE as previously described (Drug Metab Lett. 2010; 4: 139–48). Increasing amounts of 13(S)-H(P)ODE linearly increased the fluorescence readout using the Amplex Red assay as described in A.

We used Amplex Red in the presence of HRP to specifically quantify the rate of lipid peroxidation (HDLox) of a specific amount of HDL cholesterol. The Amplex Red assay could detect a concentration dependent increase in the amount of hydroperoxides associated with increasing amount of added HDL cholesterol (Fig. S4). In addition, the Amplex Red assay could reliably quantify the content of hydroperoxides associated with a specific amount of HDL cholesterol (Fig. 2). Finally, the Amplex Red could reliably measure HDLox with low interassay and intra-assay experimental variability (<11%) (Fig. S5), irrespective of the HDL isolation (Fig. S6).

### The Amplex Red assay can detect dysfunctional HDL *in vivo*

**Figure 3:**
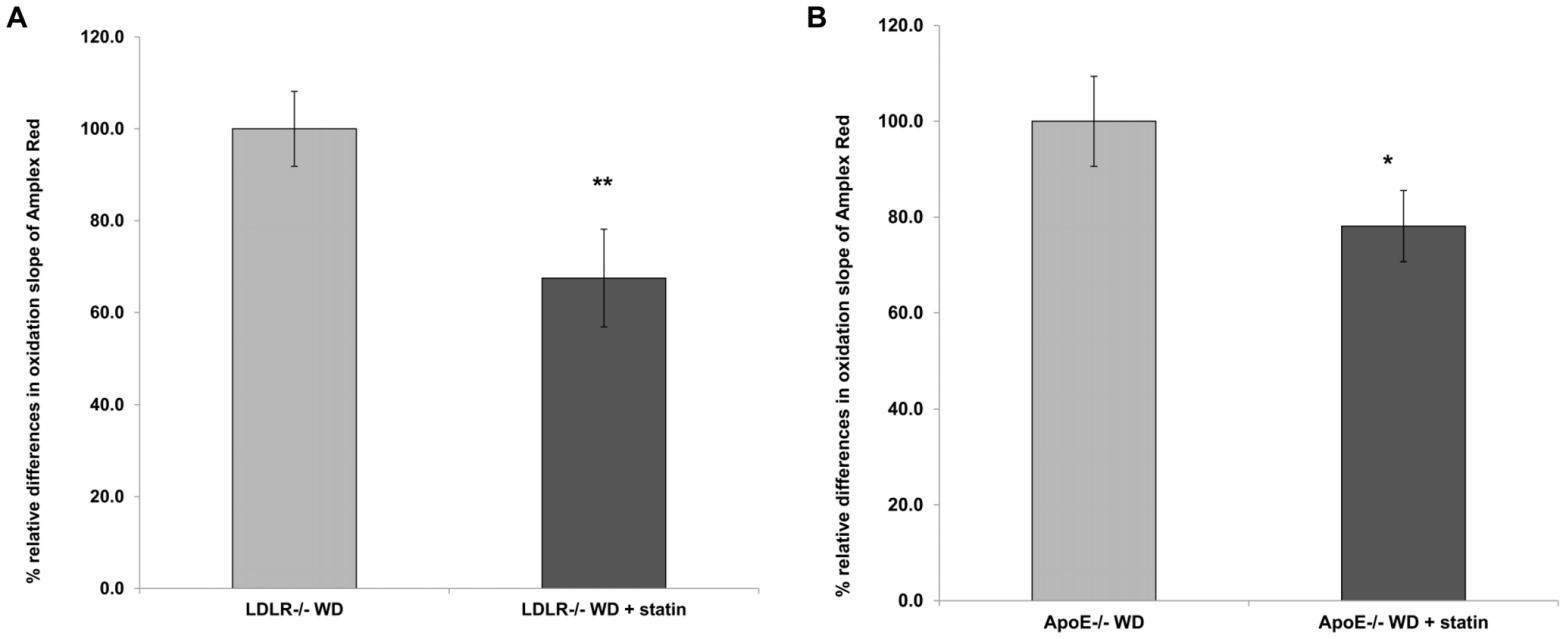
The Amplex red assay of HDL function can detect established effect of statins on functional properties of HDL in animal models of atherosclerosis. A: By using FPLC, HDL was isolated from three pooled plasma samples from LDLR^−/−^ mice on Western diet (LDLR^−/−^ WD) for two weeks and from three pooled plasma samples from LDLR^−/−^ mice on Western diet for two weeks that were also treated with pravastatin 12.5 µg/ml for two weeks. Each plasma sample was pooled from 4 mice (12 mice in total). Oxidation of Amplex Red was assessed as in Figure 2, using 2.5 µg (cholesterol) of added HDL. The oxidation slope of Amplex Red in the presence of HDL from LDLR^−/−^ WD + statin was normalized to the oxidation slope of Amplex Red in the presence of HDL from LDLR^−/−^ WD, and the percent relative differences are shown. The data represent the average of measurements from three independent experiments. There was a statistically significant reduction in the oxidation slope of Amplex Red in the presence of HDL isolated from LDLR^−/−^ WD + statin mice compared with the oxidation slope of DHR in the presence of HDL isolated from LDLR^−/−^ WD mice (** *P* = 0.01) B: By using FPLC, HDL was isolated from three pooled plasma samples from ApoE^−/−^ female mice on Western diet (ApoE^−/−^ WD) for two weeks and from three pooled plasma samples from ApoE^−/−^ female mice on Western diet for two weeks that were also treated with pravastatin 12.5 µg/ml for two weeks. Each plasma sample was pooled from 4 mice (12 mice in total). Oxidation of Amplex Red was assessed as in A. There was a statistically significant reduction in the oxidation slope of Amplex Red in the presence of HDL isolated from ApoE^−/−^ WD + statin mice compared with the oxidation slope of Amplex Red in the presence of HDL isolated from ApoE^−/−^ WD mice (** *P* = 0.01).

**Figure 4:**
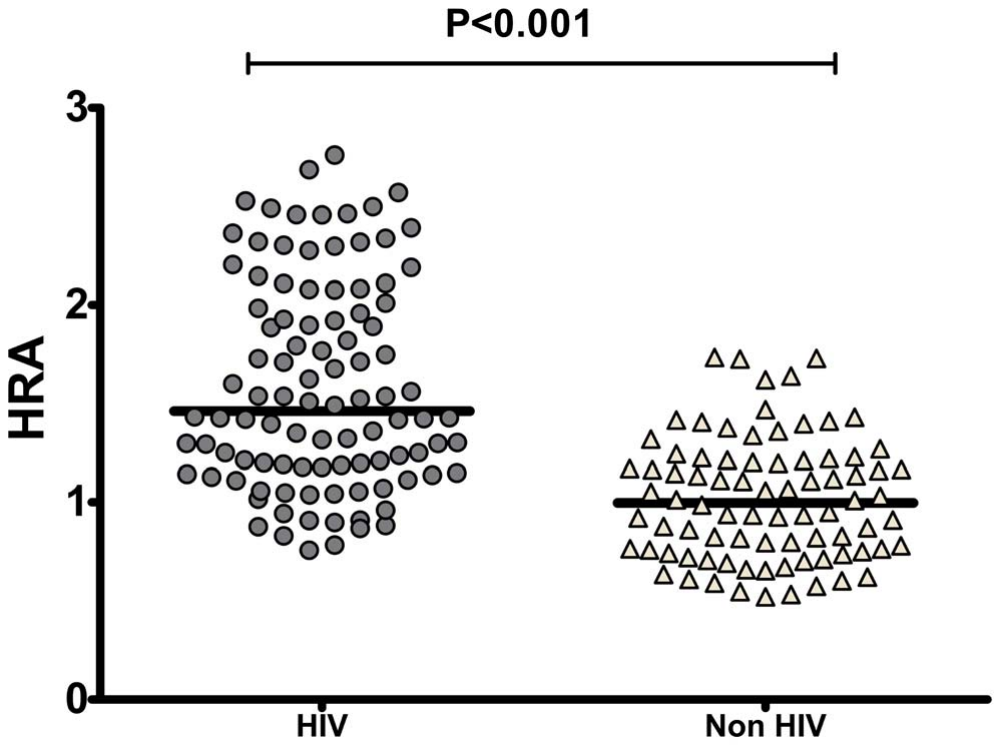
The Amplex Red Assay of HDL function can detect acute phase HDL in vivo in subjects previously shown to have dysfunctional HDL. ApoB depleted serum was isolated by PEG precipitation from 50 healthy subjects and 100 patients with HIV infection and that have previously been shown to have acute phase HDL (Lipids Health Dis 2012; 11: 87). The Amplex Red oxidation rate (AROR) as a marker of HDL redox activity (HDLox) was determined as described in Figure 2 and Figure S10. The HIV-infected subjects had significantly higher HDLox (1.59±0.53) compared to the uninfected subjects 1.01±0.31) (p<0.001).

To further validate the new method we used the Amplex Red assay to detect dysfunctional HDL in conditions in which dysfunctional HDL is known to be present such as established animal models of atherosclerosis [12], [14], and Human Immunodeficiency Virus infection (HIV) [12]. The Amplex Red assay could detect established effect of statins on functional properties of HDL in animal models of atherosclerosis such as LDLR−/− (Fig. 3a) and ApoE−/− mice (Fig. 3b). In addition, using samples from a previous cohort of HIV patients known to have dysfunctional HDL by using previously established assays of HDL function [12], [22], the Amplex Red assay confirmed that these patients had higher HDLox compared to healthy controls (Fig. 4). Thus, using this methodology we demonstrate that HDL from patients with dysfunctional HDL has a higher rate of lipid peroxidation of a specific amount of HDL (HDLox) compared to HDL from healthy patients.

### Results from the Amplex Red assay correlate with previously validated cell-based and cell-free assays

**Figure 5:**
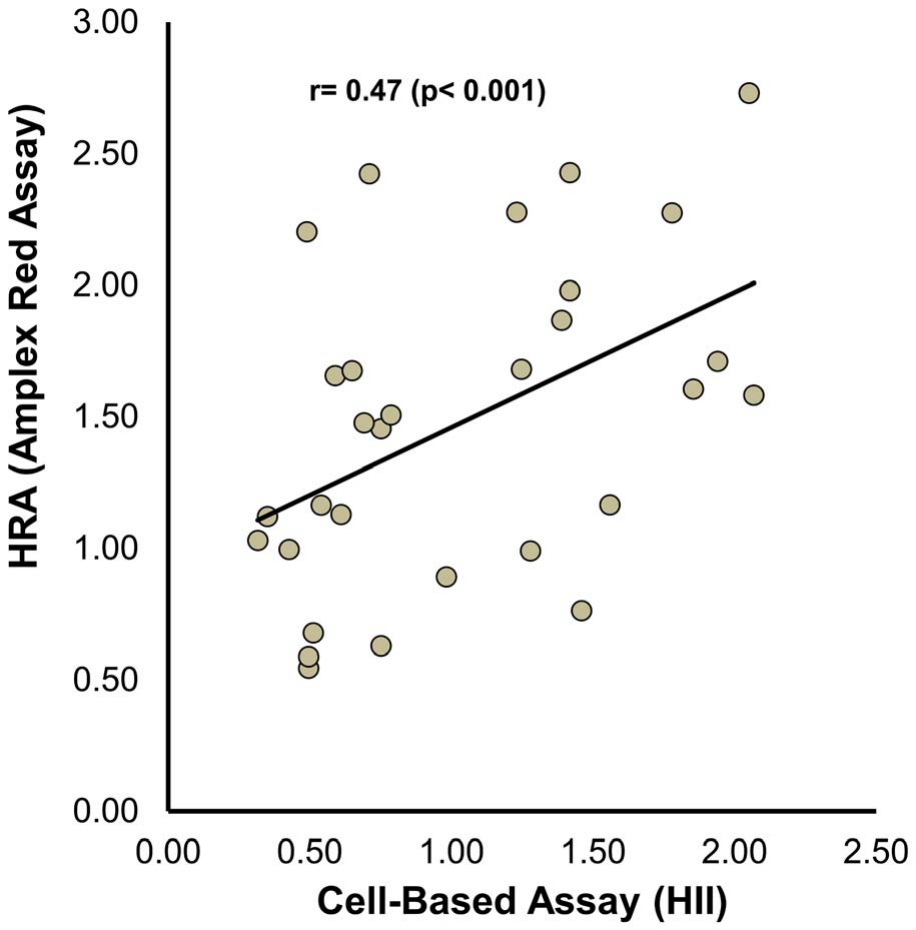
The readout from the Amplex Red Assay of HDL function correlates significantly to the readout of a previously validated cell based assay of HDL function. Thirty samples of FPLC-purified HDL were assessed for their HDL redox activity (HDLox) using the Amplex Red assay as shown in Figure 2, and their HDL inflammatory index was determined in a cell-based assay as described in Materials and Methods. The values from each assay are plotted against each other.

**Figure 6:**
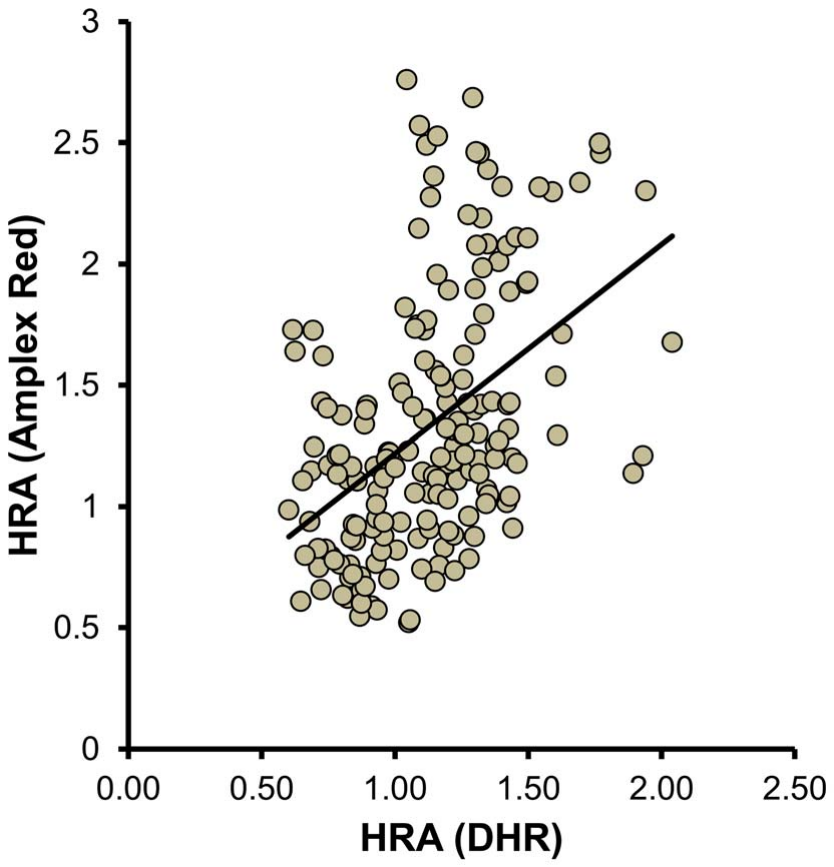
The readout from the Amplex Red Assay of HDL function correlates significantly to the readout of a previously validated biochemical cell free assay of HDL function. ApoB depleted serum was isolated by PEG precipitation from 50 healthy subjects and 100 patients with HIV infection that have previously been shown to have acute phase HDL (Lipids Health Dis 2012; 11: 87). HDL redox activity (HDLox) was determined with the Amplex Red assay as described in Figure 2 and with the dihydrorhodamine (DHR) assay as described in Methods. Non cryopreserved apoB depleted serum was used for the DHR assay and the readout was normalized by the readout of a pooled control as described in Figure S8. The values from each assay are plotted against each other (r = 0.46, p<0.001).

To compare the results of the Amplex Red assay to those obtained using a validated cell-based assay [8], [12] 30 HDL samples were assessed using both assays (Fig.5). Comparing HDLox as measured with Amplex Red to the HDL inflammatory index, there was a significant positive correlation (r = 0.47, p<0.001). Moreover, to compare the results of the Amplex Red assay to those obtained using a validated cell-free assay [12] 60 HDL samples were assessed using both assays (Fig. 6). HDLox as measured with Amplex Red correlated significantly to the HDLox as measured with DHR (r = 0.46, p<0.001).

### Standardization of the Amplex Red assay of HDL function

The lack of standardization of assays of HDL function may limit comparison of results between studies. An Amplex red stock solution of resorufin can be used to prepare a standard curve to determine the moles of product produced in the Amplex Red reaction [35], [41]. Using this approach the fluorescence readout that corresponds to HDLox can be expressed as the rate of formation of specific amount of resorfurin per specific unit of time (e.g. µM resorfurin/min) and this readout can be directly compared among different experiments especially since all the reagents used in the Amplex red assay, including all buffers, are commercially available and accessible to all researchers (Fig. S7).

We also used a control sample which is prepared from pooled HDL samples isolated from plasma blood bank specimens to standardize the assay (similarly to INR, International Normalized Ratio) as previously [42](Figs. S8, S9). Since eligibility criteria for blood bank donors are well defined worldwide [29]–[31], this approach may be used to create a universal control even when pooled controls from different blood banks are used to compare results of assays of HDL function between different studies (Fig. S8). The use of this control may also aid in controlling for unforeseen and unaccounted-for variables that may be affecting the readout including matrix effects and artifactual oxidation. Finally the Amplex Red assay can be further standardized by using the HDL concentration as determined by the clinical laboratory (a well-standardized measurement) rather than the HDL cholesterol concentration as determined by a cholesterol assay (as described in Methods) to adjust the fluorescence readout for the amount of HDL cholesterol in each sample (Fig. S10).

### Freeze-thaw can affect HDLox as measured by the Amplex Red assay

Similar to our previous results, [12], [22] we found that cryopreserved samples up to one freeze-thaw cycle can reliably be used to determine HDLox in the Amplex red assay [22] (Figure S11). The standardization method with the pooled control may minimize the effect of multiple freeze-thaw cycles on determination of HDLox using the Amplex Red assay (Fig. S11).

### Matrix effects can affect HDLox as measured by the Amplex Red assay

Similar to our previous methodology [12], [22], we have confirmed the effects of different matrices such as plasma or serum on biochemical assays of HDL function and we conclude that plasma citrate or serum should be best used to determine HDL redox activity in the Amplex red assay since heparin and EDTA can affect determination of ROS in biological fluids [43](Fig. S12). The standardization method with the pooled control tended to minimize the effect of different matrices on determination of HDLox (Fig. S12).

### Sample handling and long term cryopreservation can affect HDLox as measured by the Amplex Red assay

We then determined the effects of long-term storage of blood specimens at −80C on HDLox. We found that long-term storage of blood samples did not significantly change the ability of the assay to demonstrate relative differences in the HDLox between different groups of patients (Fig. S13). However, there was a modest increase in the HDLox of a specific amount of HDL cholesterol of the same samples that had been stored an additional year. Thus, although artifactual oxidation of protein and/or lipid components of HDL and plasma/serum may affect assays that measure oxidative state of HDL to a certain extent, the relative differences in the oxidative state of HDL between different groups can still be quantified using cryopreserved samples. Ideally, freshly isolated (not cryopreserved) HDL should be used for biochemical assays of HDL function that measure HDLox.

### The immunoaffinity capture of HDL can be used in the Amplex Red assay of HDL function to isolate HDL and minimize albumin contamination

Although PEG precipitation is a reproducible, high throughput method to extract HDL from patient serum [4], a major issue with this method is contamination of the HDL with other plasma proteins, especially albumin [22], [44], [45]. We have previously used immunoaffinity capture of HDL to study changes in the proteome associated with dysfunctional HDL [14], [18], [19]. Commercially available total HDL ELISA kits (Kit A: Genway, San Diego, CA; Kit B: Biotang Inc, Waltham, MA) can be used to capture HDL in 96-well plates when a specific volume of blood, purified HDL or apo-B depleted serum is added. Using immunoaffinity capture of HDL and 2 different methods to detect albumin content, we showed that the HDL captured on 96-well plates is largely free of albumin (Fig. S14).

### Amplex Red can reliably measure lipid peroxidation of a specific amount of HDL isolated using immunoaffinity capture of HDL

**Figure 7:**
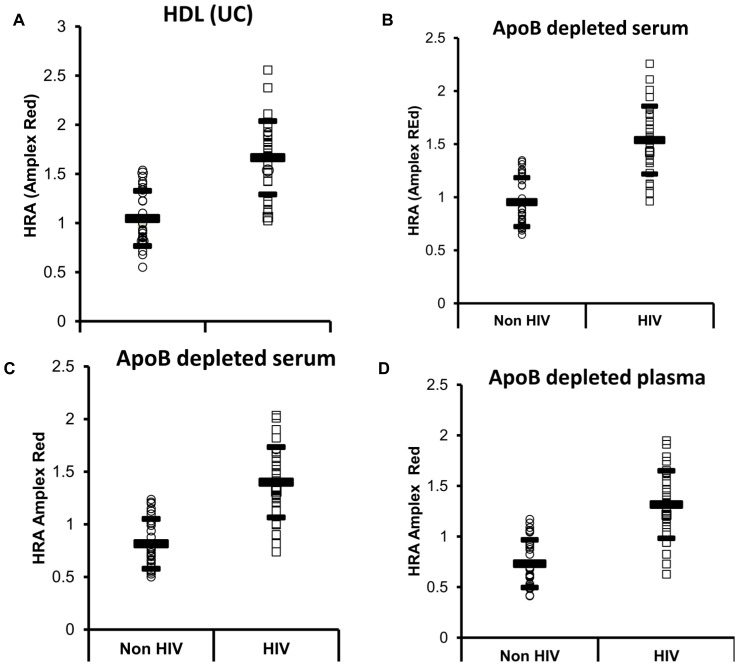
The Amplex Red Assay of HDL function in combination with immunoaffinity capture of HDL can detect acute phase HDL in vivo in subjects previously shown to have dysfunctional HDL. HDL was isolated using immunoaffinity capture as described in Methods from 30 healthy subjects and 30 patients with HIV infection that have previously been shown to have acute phase HDL (Lipids Health Dis 2012; 11: 87). The following different matrices were added in 96 well plates for immunoaffinity capture of HDL: a) purified HDL isolated by ultracentrifugation (5 µg of HDL cholesterol as determined by cholesterol assay), b) apo-B depleted serum (5 µg of HDL cholesterol as determined by cholesterol assay) c) apo-B depleted serum (100 µl) d) plasma (100 µl). In the latter two methods, the fluorescent readout (that corresponds to HDLox) was normalized to the HDL cholesterol concentration (measured by the clinical lab). ApoB depleted serum and plasma was isolated by PEG precipitation and HDL was also isolated by ultracentrifugation as described in methods. The Amplex Red oxidation rate (AROR) as a marker of HDL redox activity (HDLox) was determined as described in Figure 2 and Figure S10. The HIV-infected subjects had significantly higher HDLox (A: 1.66±0.37; B: 1.54±0.32; C: 1.40±0.33; D: 1.32±0.32) compared to the uninfected subjects (A: 1.05±0.28; B: 0.95±0.23; C: 0.81±0.24; D: 0.73±0.24) (p<0.01 for all comparisons).

**Figure 8:**
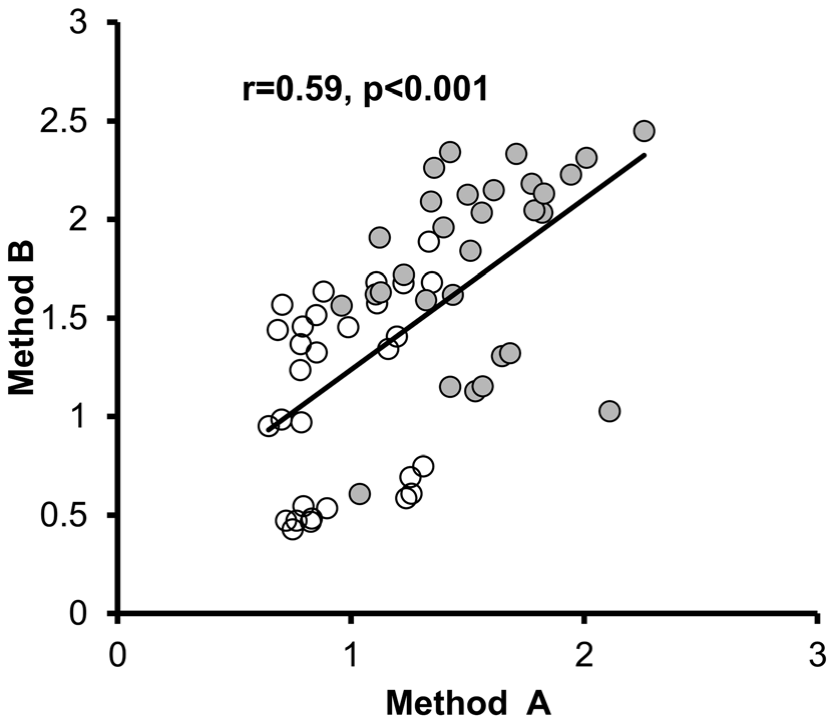
Use of different commercially available antibodies does not affect significantly the immunoaffinity capture of HDL and determination of HDLox using the Amplex Red assay. HDL was isolated using immunoaffinity capture as described in Methods and Figure 7 from 30 healthy subjects (white circles) and 30 patients with HIV infection (solid circles). Two different antibodies were used (kit A and Kit B) as described in Methods. The Amplex Red oxidation rate (AROR) as a marker of HDLox was determined as described in Figure 2 and Figure S10. The values from each assay are plotted against each other.

Using this methodology with different matrices we demonstrate that HDL from patients with dysfunctional HDL has a higher rate of lipid peroxidation of a specific amount of HDL compared to HDL from healthy patients (Fig. 7). However, since the proteome of dysfunctional HDL has not been fully elucidated [19], it is possible that dysfunctional HDL may bind less efficiently to different commercially available HDL antibodies and the total amount of HDL protein may not be directly compared between different subjects. Thus, to determine whether the approach of immunoaffinity capture of HDL depends on the quality of commercially available HDL antibodies, we used 2 different commercially available HDL kits with the Amplex Red assay (kit A and B) to measure HDLox in 60 samples. We found comparable results (Fig. 8) which indicate that the detected differences in HDLox are real and not artificial secondary to variations in binding of HDL to the antibody. Thus, the suggested method may allow high throughput isolation of HDL and in situ detection of HDLox that is associated with HDL function.

### The Amplex Red-based assay of HDLox in combination with HDL immunocapture yields reproducible measurements

To assess which commercially available HDL antibody used to capture HDL in 96-well plates gives the least experimental variability, 5 HDL samples were assessed in four independent experiments using 5 different commercially available kits (kits A–E as described in Methods). From all 5 different commercially available kits, kit B had the least experimental variability with an interassay variability ranging from 5.2 to 7.8% (mean 6.7%) and inter-assay variability ranging from 5.4 to 10.5% (mean 8.2%). Finally, from all the 4 different matrices used for HDL capture as outlined above, for a specific HDL antibody addition of a specific volume of plasma (100 µl) gave the least experimental variability, followed by addition of a specific volume of apoB depleted serum (100 µl). Thus, using specific volume of plasma as input to 96 well plates for HDL isolation, kit B for HDL capture, and normalization of the fluorescent readout (that corresponds to HDLox) by the HDL cholesterol concentration (as measured by the clinical lab) was the most reproducible method to measure HDLox.

### The HDLox as measured with the novel assay has the potential to be used as a marker of cardiovascular disease in humans

**Figure 9:**
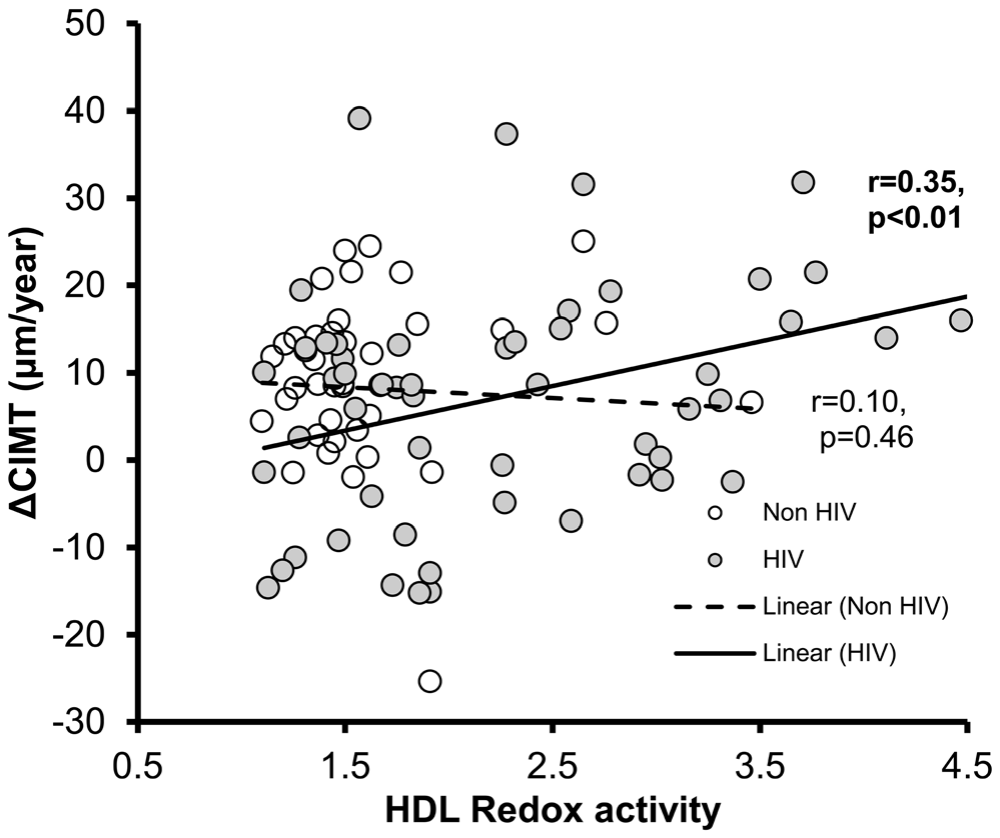
Increased HDL redox activity (HDLox), as measured by the Amplex Red Method and the immunoaffinity capture, is independently associated with progression of atherosclerosis in HIV-1- infected subjects in vivo. Scatter plot of the Rate of Change in Carotid intima–media thickness (CIMT) (ΔCIMT) and HDLox for 55 HIV-infected subjects (solid circles) and 36 uninfected controls (white circles). HDL ELISA kit was used to capture HDL in 96-well plates (kit B) as described in Methods. HDLox was determined as described in Figure 2 and Figure S10. The values from HDLox for each subject are plotted against ΔCIMT. In multivariate analysis of the HIV-infected subjects, higher baseline HDLox was associated with the ΔCIMT increasing by 2.3 mm/year (95% CI  =  (0.24, 5.6); p = 0.03) but no association between ΔCIMT and HDLox was seen in the controls (not shown).

To validate the utility of the new assay to measure HDLox as a marker of disease in humans, HDLox was measured blindly using plasma samples from a previously described cohort of 55 HIV infected subjects and 36 uninfected matched controls [28] and the Amplex Red assay. We found that HDLox was independently associated with progression of subclinical atherosclerosis in HIV-infected subjects (Fig. 9).

### The HDLox as measured with the novel assay can be used as a marker of biologic processes in humans

**Figure 10:**
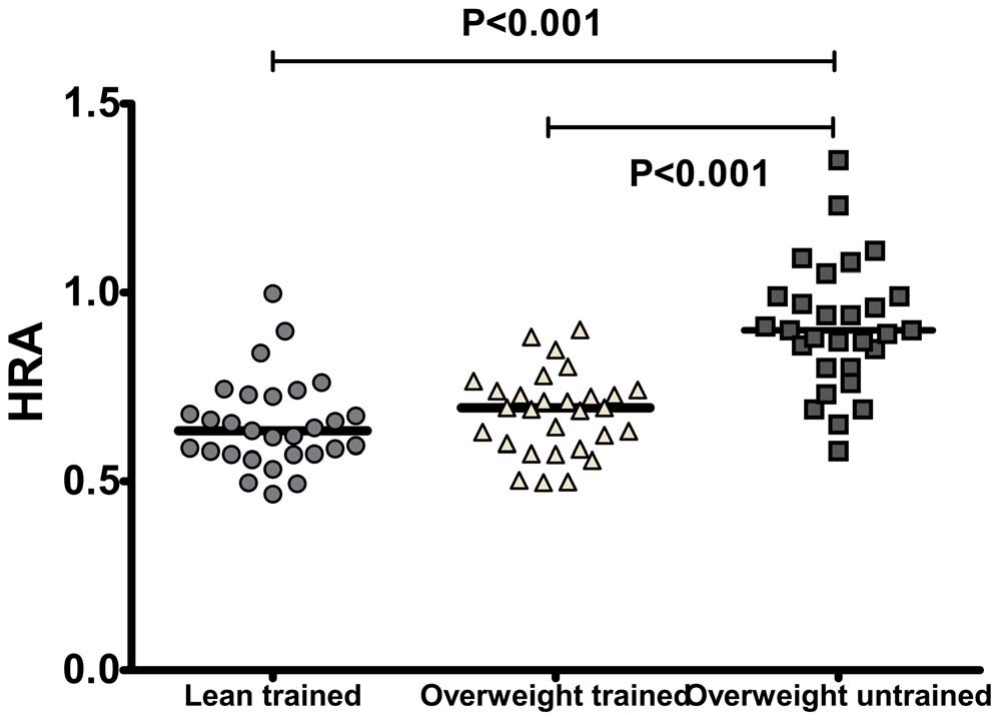
The Amplex Red assay of HDL function can detect previously established favorable effects of exercise on HDL function. HDLox was measured as described in Figure 2 and Figure S10 in a cohort of 90 humans looking into the effect of exercise on metabolic and other physiological parameters. In this study we found that high-intensity resistance training (RT) improved central and brachial blood pressures in the overweight untrained (OU) group, while having no effect on major indices of arterial stiffness in overweight/obese young men, without weight loss. Using the samples from this study we found that HDLox was significantly lower in both trained groups compared to the untrained group (LT vs. OU: 0.65±0.12 vs. 0.91±0.17, p = <0.001; OT vs. OU: 0.68±0.11vs. 0.91±0.17, p = 0.003), and LT and OT were not significantly different (p = 0.12).

**Figure 11:**
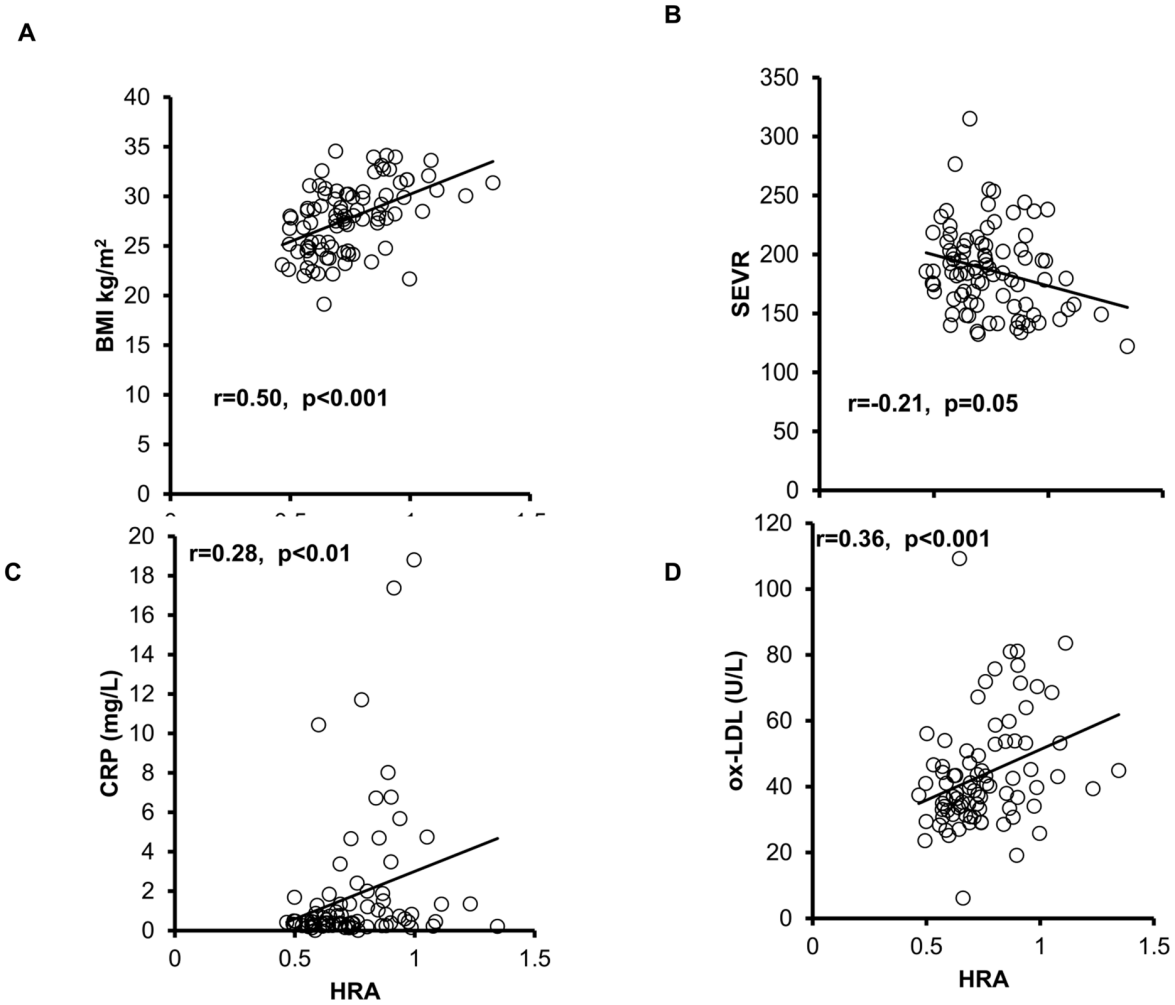
The HDLox as measured with the novel assay is significantly associated with numerous anthropometric, metabolic and physiological parameters in humans. HDLox was measured as described in Figure 2 and Figure S10 in a previous cohort of 100 humans looking into the effect of exercise on metabolic and other physiological parameters. The values from HDLox for each subject are plotted against representative physiological parameters such as Body Mass Index (BMI), subendocardial viability ratio (SEVR), a noninvasive measure of subendocardial perfusion, C reactive protein (CRP) and oxidized Low Density Lipoprotein (ox-LDL).

To further validate the utility of the new assay to measure HDLox as a marker of HDL function and other physiological processes in humans, HDLox was measured blindly in a previously established cohort of 90 subjects that looked into the effect of exercise on metabolic and other physiological parameters. Using the serum samples from this study and the Amplex Red assay of HDL function we found that exercise improved HDL function, similarly to previous studies (Fig. 10)[46], [47]. Additionally, we assessed associations of HDLox with indices of vascular and metabolic disease (Fig.11). HDLox (adjusted for HDL cholesterol concentration as outlined above) had significant negative associations with the subendocardial viability ratio (r = −0.21, p = 0.05), relative strength (r = −0.45, p<0.001), homeostatic model assessment of insulin resistance (r = −0.25, p = 0.02), HDL (r = −0.92, p = <0.00001), adiponectin (r = −0.29, p<0.01) and significant positive associations with anthropometric parameters of obesity such as BMI (r = 0.50, p = <0.0001), waist circumference (r = 0.59, p = <0.0001), trunk fat (r = 0.56, p = <0.0001), total fat mass (r = 0.55, p = <0.0001), C-reactive protein (CRP) (r = 0.28, p = <0.001), fasting glucose (r = 0.23, p = 0.03), fasting insulin (r =  0.21, p = 0.04), amylin (r = 0.23, p = 0.04), leptin (r = 0.48, p<0.001), oxidized LDL (r = 0.36, p<0.001), triglycerides (r = 0.36, p<0.001). These associations remained significant in multivariate analysis after adjusting for metabolic and anthropometric parameters. These pilot data demonstrate our ability to measure HDLox and associate cardiovascular and metabolic risk phenotypes with these measures.

## Discussion

Growing evidence suggests that HDL varies significantly in its phenotype and influence on cardiovascular disease risk [1], [11]. HDL particles are heterogeneous in shape, density, size, composition and have multiple functional properties such as reverse cholesterol transport (RCT), anti-oxidant, anti-inflammatory, and antithrombotic activities [1]. HDL are “Janus-like” lipoproteins with the capacity to be anti-inflammatory in the basal state and proinflammatory during acute-phase responses [1], [2]. Previous work has also suggested dysfunctional HDL to be pronounced in chronic inflammatory conditions that predispose to atherosclerosis [2], [10], [11], [48]. This impaired (dysfunctional) HDL, is characterized by (i) decreased levels and activity of anti-inflammatory, antioxidant factors; [1], [2] (ii) gain of pro-inflammatory proteins; [49] (iii) increased lipid hydroperoxide (LOOH) content; [50] (iv) reduced potential to efflux cholesterol; [51] and (v) diminished ability to prevent LDL oxidation [52]. Measuring the functional status of HDL, rather than HDL cholesterol concentration, may be more informative in predicting cardiovascular disease risk [53]. Unfortunately, the most widely used assays for HDL function have been cell-based assays [1] which are highly labor-intensive and subject to substantial assay variation [5]–[7] since the optimal method for performing such studies with regard to donor cells, type of acceptor and type of readout has yet to be determined. Thus, cell based assays of HDL function are technically prohibitive, especially for use in large-scale clinical studies.

Cell-free assays of HDL function may be more precise than cell-based assays because they measure a biochemical rather than biologic phenomenon. Thus, we previously developed a cell free assay based on the fluorochrome DCF that assessed the HDL inflammatory index (HII), a measure of the capacity of HDL to prevent the formation or to inactivate oxidized phospholipids produced by LDL [9]. This was based on our demonstration that levels of ROS (such as lipid hydroperoxides produced from oxidation of lipoproteins) are significantly associated with inflammatory properties of HDL that are measured by the HII [13]. This assay has been previously used in multiple studies in humans [13], [54], [55] and this measurement of HDL has been associated with other measures of HDL function such protein biomarkers associated with dysfunctional HDL [3], [4], [14]–[19], HDL anti-inflammatory function measured as the ability of test HDLs to inhibit LDL-induced monocyte chemotactic activity in human aortic endothelial cell monolayers [14], [17], [19] and measurement of oxidized fatty acids in HDLs [15], [17], [56]. It has also been validated *in vivo* in animal models of atherosclerosis [15]. Furthermore, HDL function measured using this assay has been correlated with systemic inflammation in coronary heart disease (CHD) patients [16], [18] and cardiovascular disease outcome [4], [55]. However, the oxidative instability of DCF-DA, variations in the donor LDL used in the assay and increased lipid-probe interactions of two lipoproteins used in the same biochemical reaction increased experimental variability of this method [18]. Thus, we have recently developed an assay of HDL function that is based on DHR, a more stable fluorochrome, in which the endogenous HDL redox activity is assessed in terms of the capacity of a specific amount of HDL cholesterol to engage *in vitro* redox cycling [57]. However, although this assay was validated *in vitro* [12] and in *vivo* [12], [58]–[60], due to certain biochemical limitations of the DHR assay that have previously been described in detail [12], [22], only fresh apoB depleted serum samples and purified HDL isolated by ultracentrifugation may be used with the DHR assay and this may limit its utility for large scale clinical studies.

Recent interest has focused on the functional consequences of HDL oxidation. High density lipoprotein is the major carrier of lipid hydroperoxides in human blood plasma [34], [35] We have previously shown that increased lipid hydroperoxide (LOOH) content is associated with dysfunctional HDL [51], [61] and that oxidized HDL is dysfunctional HDL [62]–[64]. Dysfunctional HDL has increased endogenous “ROS load” and redox activity [65]. Oxidation could conceivably contribute to the formation of dysfunctional HDL, proposed to be present in humans with cardiovascular disease [12]. One potentially important pathway for generating dysfunctional HDL via oxidation involves myeloperoxidase that mediates conversion of protein tyrosine residues to 3-chlorotyrosine, and methionine residues to methionine sulfoxide (MetO) [66]. In addition, MetO can also be formed from exposure of HDL's major protein, apolipoprotein A-I (apoA-I) to H_2_O_2_ [67] or lipid hydroperoxide [68], the latter generated during the oxidation of HDL lipids. Reactive oxygen species (ROS) such as 1*e*-oxidants (i.e., hydroxyl radical and metal ions) have previously been shown to oxidize tyrosine and methionine residues which can have dramatic consequences on the functions of apoA-I/HDL, including reverse cholesterol transport [69].

Herein we describe an approach to quantify the HDL redox activity, a measure of HDL function, in a novel cell-free, biochemical assay. We looked for a fluorescent probe that would meet the following criteria i) would reliably and specifically quantify the rate of lipid peroxidation of a specific amount of HDL cholesterol ii) enzymatic amplification of the measurement of ROS with this probe would overcome lipid-probe-ROS interactions that may be a limitation of fluorescent biochemical assays of HDL function [12], [22] iii) would be a reasonable choice to measure ROS accurately based on the known challenges and limitations that most widely used fluorescent probes have for detecting and measuring ROS [22].

Amplex red is a fluorogenic substrate with very low background fluorescence, which reacts with H_2_O_2_ with a 1∶1 stoichiometry to produce highly fluorescent resorufin [70]. Amplex Red can be oxidized by HRP which vastly increases the yield of resorufin [35], [41] and this assay is a reliable method to continuously measure the extracellular formation of H_2_O_2_ [35], [41] in 96 well plate format [35], [41]. We have previously used the Amplex Red reagent to quantify ROS produced by oxidized lipids in cell culture supernatants [34], [35]. Indeed, Amplex Red in the presence of the enzyme cholesterol oxidase has been reliably used to quantify cholesterol content of HDL based on lipid peroxidation of HDL [71]. Using a modification of this well described assay (Fig.1), we demonstrate that Amplex Red, in the absence of cholesterol oxidase and for the same amount of HDL cholesterol, can detect differences in the rate of lipid peroxidation between different HDL samples that correspond to differences in HDL function.

This approach in combination with immunoaffinity capture of HDL for HDL isolation may overcome many confounding factors that affected the readout in the previously described cell free assays of HDL function and provides a measurement of HDLox that correlates well with previously validated cell-based and cell-free assays of HDL function. Because a large proportion of cholesterol in blood is in the form of cholesteryl esters, cholesterol esterase is used to produce free cholesterol from cholesterol esters [34], [35]. In addition the assay may be modified and cholesterol esterase may or may not be added in the Amplex Red reagent so that peroxidation of HDL cholesterol in the form of cholesteryl esters versus free cholesterol can be determined [34], [35].

This Amplex Red-based cell-free assay improves upon the prior DHR-based cell-free assay. While also measuring the HDLox the biochemistry of the Amplex Red fluorochrome and its ability to detect lipid hydroperoxides is well established compared to DHR [12], [22]. In the absence of cholesterol oxidase the Amplex Red detects the intrinsic hydroperoxide content of a specific amount of HDL cholesterol and in the presence of HRP may overcome previously described lipid-probe interactions [22]. In addition, the Amplex Red -based assay improves upon the prior DCF-based assay [13]. While DCF may also measure lipid peroxidation [13], [20], [21], this earlier assay had several technical challenges that have limited its utility. DCFH-DA is unstable and needs to be dissolved in methanol for activation to the active molecule DCFH which is prone to auto-oxidation and needs to be protected from room air [41], [72]. In contrast, Amplex Red is relatively stable, and oxidizes at a predictable rate in the presence of HRP when exposed to room air [41]. Furthermore, conversion of DCFH-DA to DCFH can be mediated by esterases that are carried over variably during the lipid purification process, adding another variable that is difficult to control, while Amplex Red requires no activation and is not prone to this effect [12], [22], [41]. Finally, different diluents for biochemical assays of HDL function may affect the oxidation rate of the indicator compound; this may also explain the variability observed in the DCFH-DA assay between different research groups [12]. In contrast, all the reagents used in the Amplex Red assay are commercially available and thus this assay can be better standardized. Moreover, the use of immunoaffinity capture may allow HDL isolation and use of this method in large scale studies and removal of much of the albumin bound to the HDL particle that may alter the association of ROS with lipoproteins [12], [44], removes lipid peroxidation products [45] and can also significantly interfere with the fluorescent readout in biochemical assays of HDL function [22]. Finally, the inter-assay variability of <15% compares favorably with cell-based assays of HDL function which have variability of >15% [44].

In addition using animal models of atherosclerosis and 2 different human studies we show that this assay can be used as a potential marker of cardiovascular disease and biologic processes in vivo. The correlation of HDLox as measured by the Amplex Red assay to the biologic readout of HDL in a cell-based assay is consistent with a proposed mechanism whereby HDL exerts its effects through modulating ROS [10], [11], and suggests that the assay accurately reflects HDL function. Further support for the biological relevance of this measurement is the finding that for the same amount of HDL cholesterol the HDLox was significantly reduced in HDL from statin treated mice with atherosclerosis compared to HDL from non-statin treated mice. Indeed, treatment of these mice with statins has previously been shown to reduce inflammatory properties of HDL [13] and we demonstrate that the Amplex Red assay can detect the favorable effect of statins on functional properties of HDL. In the pilot human studies described in this manuscript we demonstrate the ability of the Amplex Red Assay to measure HDLox and associate cardiovascular and metabolic risk phenotypes with these measures of HDL function. For example we confirmed previous data that HDLox is associated with anthropometric parameters of obesity in humans and cardiovascular disease in HIV-1 infected subjects [3], [4], [12], [58]–[60]. Moreover, the Amplex Red assay can detect dysfunctional HDL in patients with HIV infection confirming our previous results [32]. The fact that our assay quantifies HDLox and that the HDLox levels correlate with surrogate measures of cardiovascular disease and other physiological parameters in humans such as obesity and insulin resistance increases its applicability to biological samples, at least in the context of cardiovascular diseases. This is because 1*e*-oxidants (i.e., hydroxyl radical) contribute to oxidative modifications taking place in the affected arterial wall during atherogenesis [12], [22]. Thus, while “oxidized HDL” is not a chemically defined term, the oxidation rate of Amplex Red corresponds to the intrinsic HDLox of specific amount of HDL lipoproteins. However, we did not detect a significant association between HDLox and progression of subclinical atherosclerosis in a small group of healthy subjects. This may have been explained by the relative small size of our pilot study and the use of cryopreserved samples, especially since the anti-oxidative capacity of HDL has been studied only in association with clinically significant coronary artery disease in larger studies [4], [55], [59], [60]. However, in our exercise study of HIV uninfected subjects with no known cardiovascular disease we did show, for the first time to our knowledge, that HDLox correlated significantly with subendocardial viability ratio (SEVR), a noninvasive measure of subendocardial perfusion, C reactive protein (CRP) and oxidized Low Density Lipoprotein (ox-LDL) which is associated with the metabolic syndrome [58], [73]. Larger case control studies will further define the utility of this assay as a marker of biologic processes and diseases in humans.

Finally, we showed that matrix effects, freeze thaw, sample handling and long term storage of blood specimens at −80C can affect the HDLox but the relative differences in HDLox between different samples can still be reliably detected with the Amplex Red reagent, in either serum or non EDTA plasma, fresh or cryopreserved samples, and with up to 2 freeze-thaw cycles. We also demonstrated that the HDLox readout can be normalized by the HDL cholesterol amount and the assay can be standardized using either an Amplex red stock solution of resorufin or a control sample which is prepared from pooled HDL samples isolated from plasma blood bank specimens. Since HDL is a lipoprotein and under inflammatory conditions, it has been shown that both the protein and phospholipid moieties of HDL are substantially altered, thereby modifying the functional characteristics of the HDL particles [1], [2], normalization of the readout of assays of HDL function by HDL protein content (determined by a BCA protein assay) [14], [74] or plasma apolipoprotein A-I (apoA-I; determined by ELISA) content has been suggested [20], [75]. Normalization of the fluorescence readout (HDLox) by apoA-I levels did not significantly affect the differences in HDLox between HIV infected and uninfected groups compared to not normalizing. However, the oxidative modifications occurring to HDL in the diseased artery wall are conceivably more complex than *in vitro* [76] and HDL is subject to continuous remodeling *in vivo* [10], [11], [76]–[78]. This includes dissociation of apoA-I from the lipoprotein particle, a process that could be increased by oxidation [10], [11], [76]–[78] so it is unclear if the HDLox readout should be normalized by apoA-1 levels or HDL protein levels especially since the proteome of dysfunctional HDL has not been fully elucidated [19]. Clearly, future studies are required to assess these various aspects.

In conclusion, this new assay offers a rapid method for measuring the redox properties of HDL. It yields results that correlate well with previously validated cell-based and cell-free assays of HDL function and can be used as a potential marker of cardiovascular disease and biologic processes in humans. This new technical approach may offer a convenient tool for studies of the role of HDL functional phenotype in the development of atherosclerosis *in vivo*.

## Supporting Information

Figure S1 **Flow diagram of Assay.**
(TIF)

Figure S2 **Without HRP lipid probe interactions are present but increasing amounts of HRP can increase the efficiency of detection of hydroperoxides carried by a specific amount of HDL cholesterol.** In a 96 well flat bottom plate, 50 ul of 1X reaction buffer was added to each well alone or with 5 µg (cholesterol) of apoB depleted serum (as determined by a cholesterol assay) from a donor with anti-inflammatory HDL (HDL) and from a donor with acute phase HDL (AP-HDL), each in quadruplicates. 50 µl of HRP (0.5–4 U/ml) was then added to all wells followed by incubation at 37°C for 60 min. 50 µl of Amplex Red Reagent (final concentration 300 µM) was then added to each well for a total volume of 150 µl and the rate of production of resorufin was followed at 37°C in one-minute intervals for 60 minutes. The rates of change in fluorescence between 0 and 60 minutes are plotted for the quadruplicates, as well as means/standard deviations. In the absence of HRP, fluorescence quenching and lipid-probe interactions lead to reduction in the fluorescence readout after addition of a specific amount of HDL cholesterol compared to the fluorochrome alone, consistent with our previous observations with other fluorochromes such as DHR and DCF. Addition of ≥2 U/ml of HRP lead to a specific amplification of the quantification of the hydroperoxides associated with a specific amount of HDL cholesterol. A representative sample from each type of HDL (HDL vs AP-HDL) is shown and similar results were observed for 5 different other samples (5 HDL and 5 AP-HDL). In addition similar results with HRP were observed when 5 µg of HDL cholesterol isolated by FPLC or ultracentrifugation were added to the reaction.
(TIFF)

Figure S3 **Oxidation of Amplex Red and effect of added HDL.** In a 96 well flat bottom, 50 µl of 1X reaction buffer (0.5 M potassium phosphate, pH 7.4, 0.25 M NaCl, 25 mM cholic acid, 0.5% Triton X-100) was added to each well alone or with 5 µg (cholesterol) of apoB depleted serum (as determined by a cholesterol assay) from a donor with anti-inflammatory HDL (HDL) and from a donor with acute phase HDL (AP-HDL), each in quadruplicates. 50 µl of HRP was then added to all wells followed by incubation at 37°C for 60 min. 50 µl of Amplex Red Reagent (final concentration 300 µM) was then added to each well for a total volume of 150 µl. The rate of production of resorufin was followed at 37°C in one-minute intervals using a fluorescence microplate reader set to detect 530/590 nm excitation/emission. A. The means and standard deviations of the quadruplicate fluorescence measurements are plotted over time. B. The rates of change in fluorescence between 0 and 60 minutes (calculated by linear regression) are plotted for the quadruplicates, as well as means/standard deviations. The background fluorescence of the blank well (no HDL) was subtracted from the readout of each well for each timepoint.
(TIF)

Figure S4 **The Amplex Red assay can detect a concentration dependent increase in the amount of hydroperoxides associated with increasing amount of added HDL cholesterol.** HDL isolated by ultracentrifugation was added in varying concentrations (cholesterol) to 300 µM Amplex Red in a 96 well flat bottom plate and the rate of change in fluorescence was measured as in Fig. 2 in the presence of 4 U/ml of HRP. The rates of change in fluorescence (means and standard deviations) are plotted against the amounts of added HDL. In addition similar results with HRP were observed when HDL cholesterol isolated by PEG precipitation was added to the reaction. There was a concentration dependent increase in the fluorescent readout with increasing amount of added HDL cholesterol in the presence of HRP in contrast to a concentration dependent decrease in the readout with increasing amount of added HDL cholesterol with other fluorescent probes (DCF and DHR).
(TIFF)

Figure S5 **Low inter-assay variability between measurements of HDL effects.** Rate of oxidation of Amplex Red in the presence of six different samples of HDL isolated by PEG precipitation from 6 subjects (3 HIV infected subjects with AP-HDL and 3 healthy subjects with normal HDL) was assessed as described in Fig. 2, using 5 µg (cholesterol) of added HDL. The data (means of quadruplicates) from four independent experiments are plotted. The mean inter-assay variability for these six samples was 8.6% (range 4.9 to 9.7%), and the mean intra-assay variability was 4.6% (range 2.9–7.2%). When HDL from the same subjects was isolated by UC (not plotted) the mean inter-assay variability for these six samples was 10.6% (range 6.9 to 12.6%), and the mean intra-assay variability was 6.8% (range 4.8–9.1%). In addition, measurement of the rate of oxidation of Amplex Red over 60 min lead to reduced experimental variability compared to endpoint measurement of fluorescence at 60 min.
(TIFF)

Figure S6 **Correlation of effect of HDL on Amplex Red oxidation using different methods of HDL isolation.** HDL was isolated by ultracentrifugation or PEG precipitation from 5 HIV infected patients known to have acute phase HDL (AP-HDL; shown in solid black circles) and 5 patients with normal HDL (shown in white circles). 5 ug of HDL cholesterol was then added to 300 µM Amplex Red in a 96 well flat bottom plate and the rate of change in fluorescence was measured as in Fig. 2 in the presence of 4 U/ml of HRP. The mean rates of change in fluorescence are plotted.
(TIFF)

Figure S7 **Commercially available resorufin standards can be used to standardize fluorescence-based quantification of the hydroperoxide content of a specific amount of HDL cholesterol.** A commercially available resorufin fluorescence reference standard can be used to prepare a standard curve to determine the moles of fluorescent product produced in the Amplex Red reaction according to the manufacturer's instructions. Endpoint measurement of the fluorescence signal that corresponds to production of resorufin and oxidation of the Amplex Red reagent was performed at 60 minutes as described in Fig. 2. The reference 2 mM resorufin standard was diluted accordingly to generate a standard curve of resorufin that would “fit” the dynamic range of the measured fluorescence at 60 minutes for the specific assay. Towards this end, the amount of the added cholesterol and the time of the reaction for certain photomultiplier sensitivity needs to be titrated carefully. The triplicate fluorescence readings for each standard were averaged and the mean fluorescence was calculated. The average fluorescence of the blank sample (Amplex Red alone without HDL) was subtracted from all the standards and samples and the adjusted fluorescence was calculated. The adjusted fluorescence of the standards was plotted as a function of the concentration of the resorufin standards. An example of a standard curve with a dynamic range 15.625–500 nM and six standards is shown. The fluorescence of the HDL samples was calculated in the presence of 5 µg (cholesterol) of added HDL. HDL was isolated by PEG precipitation from HIV infected subjects with acute phase HDL (AP-HDL) and healthy subjects with normal HDL. The means of quadruplicates were calculated (adjusted fluorescence). The amount of produced resorufin for each HDL sample was calculated using the equation obtained from the linear regression of the standard curve substituting adjusted fluorescence values for each sample. 2 representative samples (one with normal HDL [dashed line] and one with AP-HDL [solid line]) are shown. Since the resorufin standards are run in parallel with the samples over 60 minutes the slope of change in fluorescence per min (FU/min) can be converted into the slope of nM of resorufin produced per min (nM/min). Thus, using this approach, the resorufin concentration that is produced as a result of the specific oxidation of the Amplex Red Reagent by the hydroperoxides present in a specific amount of each HDL cholesterol sample, can be measured and can be used as a surrogate measure of the HDL redox activity and HDL function.
(TIFF)

Figure S8 **The fluorescence readout of the Amplex Red assay of HDL function can be normalized against the readout of a specific amount of HDL cholesterol isolated from pooled apoB depleted serum of healthy subjects.** A) The Amplex Red oxidation rate (AROR) was determined as described in Fig. 2 after adding 5 µg of apoB depleted serum (isolated by PEG precipitation) from 50 cryopreserved serum blood bank specimens from healthy subjects. The values represent means of triplicate samples. There was an approximately 3-fold difference between the lowest and highest AROR value (median 155, IQR 119–180 FU/min; range 74–246 FU/min). B) The 50 HDL samples were pooled in groups of five samples (pentads; 0.5 µg of HDL cholesterol from each sample) so that the total amount of HDL cholesterol in each pooled sample (pentad) was 5 µg, for a total of 10 pentads. Then the pentads were combined in various combinations and different number (5, 10, 15, 20, 25, 30, 35, 40, 45, 50) of HDL samples so that the total amount of HDL cholesterol at each pooled sample would be 5 µg. The AROR was determined as described in Fig. 2. The values represent means of triplicate samples. Using this methodology there was approximately a 2-fold reduction in the variability of determination of the AROR (for the same amount of HDL cholesterol) in healthy subjects (median 153, IQR 141–166 FU/min; range 120–186 FU/min). Thus, to correct for interassay variability across different plates and to standardize the assay, a pooled HDL control sample from at least 30 healthy volunteers may be included in each plate, AROR can be determined and sample fluorescence values may be normalized by this pooled value. The individual normalized AROR (nAROR) [nAROR  =  (AROR/AROR control] is evaluated as a ratio to the AROR of a control HDL isolated from the pooled serum. Thus, using this method we avoid expression of results of AROR in arbitrary units (FU/min) and results between different studies can be comparable provided the same pooled control is used.
(TIFF)

Figure S9 **A specific amount of HDL cholesterol isolated from pooled blood bank specimens of healthy subjects can be used as a universal control to standardize the Amplex Red assay of HDL function.** HDL was isolated using PEG precipitation from 3 different groups (A, B, C; each 30 samples) of cryopreserved serum blood bank specimens. The HDL samples in each group were pooled as described in Fig. S6 (three different blood bank pools). The Amplex Red oxidation rate (AROR) was determined as described in Materials and Methods. The mean AROR among the 3 different blood bank pools was comparable. Thus, this current approach may be used to create a universal control for determination of DOR by combining HDL samples from at least 30 different donors.
(TIFF)

Figure S10 **The HDL concentration as determined by the clinical laboratory can be used to adjust the fluorescence readout for the amount of HDL cholesterol in each sample in the Amplex Red assay of HDL function.** ApoB depleted serum was isolated by PEG precipitation from 20 subjects (10 healthy and 10 with HIV infection and acute phase HDL). The Amplex Red oxidation rate (AROR) was determined as described in Fig. 2 and HDL was added using two different methods (A and B). In method A the HDL cholesterol concentration of each sample was determined using a cholesterol assay as described in the Methods section and then 5 µg of HDL cholesterol was added to each well. The individual normalized AROR (nAROR) [nAROR  =  (AROR/AROR control] is a measure of the HDL redox activity (HRA) and is evaluated as a ratio to the AROR of a control HDL isolated from pooled serum as described in Fig. S7. In Method B the HDL cholesterol concentration of each sample (mg/dl) was measured by the clinical lab and this value is routinely available in the setting of standard clinical care. A specific volume of apoB depleted serum (50 µl) was added to each well, the AROR for each sample was determined as above and this readout was normalized by the HDL cholesterol concentration of each sample (n_HDL_AROR). A control HDL sample was created after pooling equal volumes of apoB depleted serum from 30 healthy blood bank serum. The HDL concentration of this pooled HDL control was calculated from the HDL concentrations of the individual samples (measured in mg/dl by the clinical lab) and the fluorescence readout was normalized by this value (n_HDL_AROR control). The individual normalized to control AROR is evaluated as a ratio to the AROR of a control HDL isolated from pooled serum [nAROR  =  (n_HDL_AROR/n_HDL_AROR control]. The values represent means of triplicate samples and the correlation coefficient is shown. Data from healthy subjects are shown as white circles and data from HIV infected subjects are shown as gray circles.
(TIFF)

Figure S11 **The standardization method with the pooled control minimizes the effect of multiple freeze-thaw cycles on determination of HDL redox activity (HRA) using the Amplex Red assay.** Oxidation rate of Amplex Red (AROR) in the presence of 20 different samples of HDL isolated by PEG precipitation from heparin plasma [10 from patients with Human Immunodeficiency virus (HIV-1) infection and 10 from healthy volunteers (Non HIV)] was assessed as described in Methods and in Fig. 2, using 2.5 µg (cholesterol) of added HDL. The AROR of each sample was determined within 6 hours after collection of the blood specimen and after 1–5 freeze-thaw cycles. The values represent means of triplicate samples. The % relative HRA of each HDL sample after each extra freeze-thaw cycle (for up to 5 cycles) was significantly higher (paired t-test p<0.05 for all datapoints) compared to the HDL sample that was isolated within 6 hours. Although the HRA values tended to significantly increase after each extra freeze-thaw, their correlations with the HRA value from the HDL sample that was isolated within 6 hours remained statistically significant (p<0.05 for all data points). In addition, the individual normalized AROR was evaluated as a ratio to the AROR of a control HDL isolated from pooled serum as described in Figs S7 and S9. The control HDL matched the freeze-thaw cycles of the respective HDL samples (for example if the samples were thawed once the pooled control was made from HDL samples that were thawed once, etc). This standardization method improved the correlations of the relative HRA values with the HRA value from the HDL sample that was isolated within 6 hours and tended to minimize the effect of multiple freeze-thaw cycles on determination of HRA.
(TIFF)

Figure S12 **The standardization method minimizes the effect of different matrices on oxidative properties of HDL.** Oxidation rate of Amplex Red (AROR) in the presence of 13 different samples of HDL [7 from patients with Human Immunodeficiency virus (HIV-1) infection and 6 from healthy volunteers (Non HIV)] isolated by PEG precipitation from heparin plasma, citrate plasma and serum was assessed as described in Methods and in Fig. 2. The values represent means of all the samples. The HRA values from plasma citrate samples correlated significantly (p<0.01) with the HRA values from serum samples but heparin interfered with the readout. In addition, the HRA of each sample was normalized by the HRA value of the control sample and the % relative HRA was determined as in Fig. S10. The suggested standardization method improved the correlations of the HRA values and tended to minimize the effect of different matrices on determination of HRA.
(TIFF)

Figure S13 **Long term storage of blood specimens tends to increase HDL redox activity (HRA) as determined by the Amplex Red assay but the results are comparable between different timepoints.** The Multicenter AIDS Cohort Study (MACS) has defined a group of men who remained HIV-1- seronegative despite hundreds to thousands of high-risk sexual exposures in the 1980s. The MACS cohort recruited men in 1985 for natural history studies (Am J Epidemiol 126: 310-8), and has continued to follow subjects every 6 months to the present. Using 9 stored serum samples from this cohort that were stored for 27 and 28 years at −80°C, we determined the effect of long term storage at −80C on HDL redox activity (HRA) as described in Fig. 2. The readout of each sample was expressed as % relative to the average readout of all 9 samples at 27 years of cryopreservation. The HRA as determined by the Amplex Red assay significantly increased after cryopreservation for one extra year (125±41% vs 100±31%, p value for paired t test = 0.04) and the readouts from the 2 groups correlated significantly (r = 0.69, p<0.01).
(TIFF)

Figure S14 **HDL isolated using immunoaffinity capture of HDL is largely free of albumin.** 50 ul of plasma (n = 10) was added into 96 wells and was isolated using immunoaffinity capture of HDL according to the manufacturer's instructions (Kit A). After 5 washes, 300 µl of albumin bromocresol green reaction (BCG) reagent (Thermo Scientific Inc) were added to each well and after 90 second incubation at 37°C the optical density at 630 nm was read. Results are expressed as % relative value compared to the positive control (50 µl of plasma). The median relative albumin content bound to HDL was 0.90% with the BCG method, one of the most sensitive and specific methods to detect albumin. The minimal detection of HDL-bound albumin (<0.5% relative to the positive control) was also confirmed with a secondary antibody against albumin conjugated to horseradish peroxidase (HRP) (Pierce Inc). Similar results were obtained with Kit B.
(TIFF)

Table S1 **Outline of protocol used to measure HDL lipid peroxidation (PEG method for HDL isolation).**
(DOCX)
