# Supplementary figures and images for: A High Throughput Biochemical Fluorometric Method for Measuring Lipid Peroxidation in HDL
Source: PLoS One. 2014 Nov 4;9(11):e111716. doi: 10.1371/journal.pone.0111716 (PMC4219769; doi:10.1371/journal.pone.0111716)

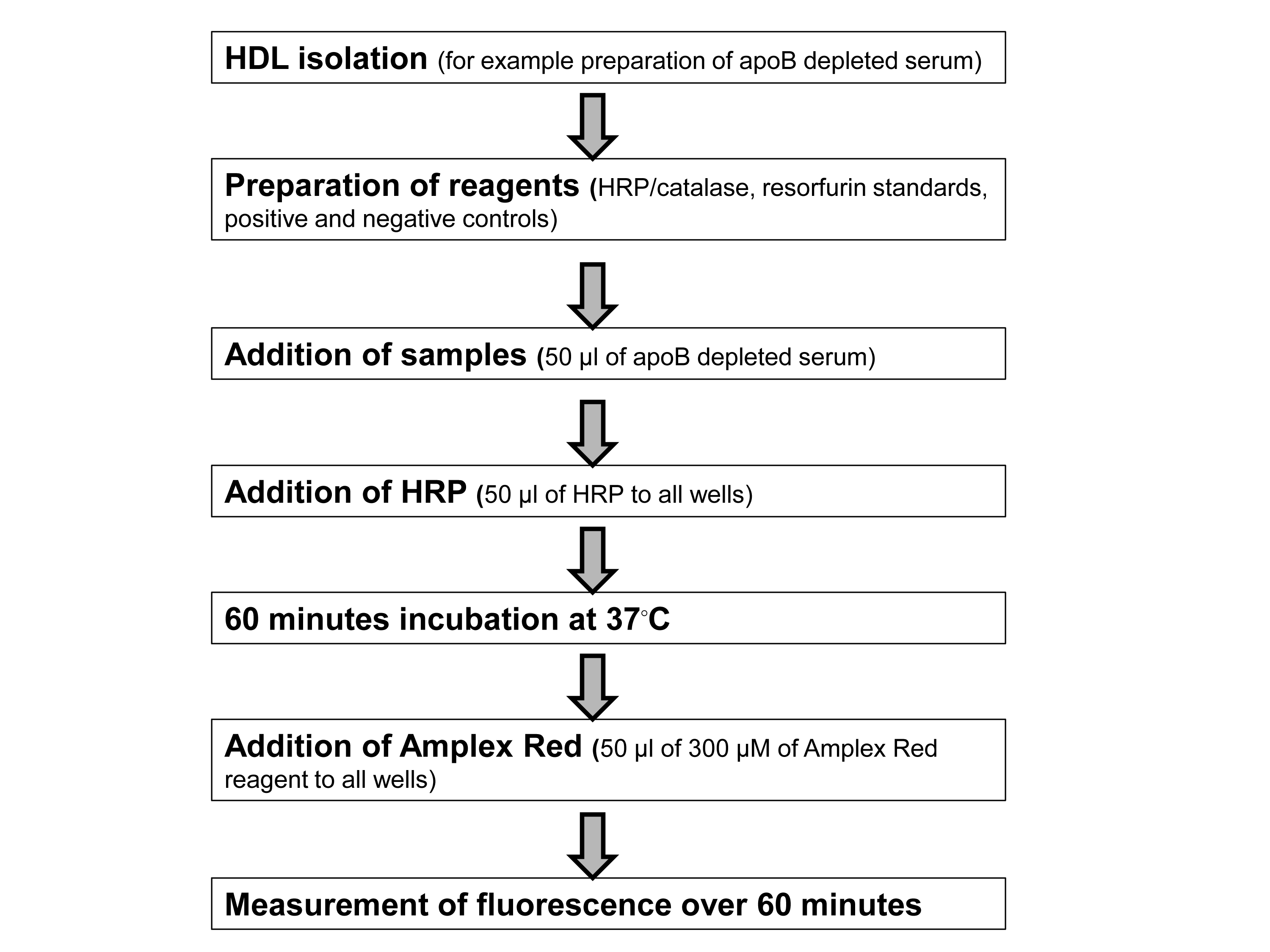

Supplement: Figure S1 — Flow diagram of Assay. (TIF) [file pone.0111716.s001.tif]

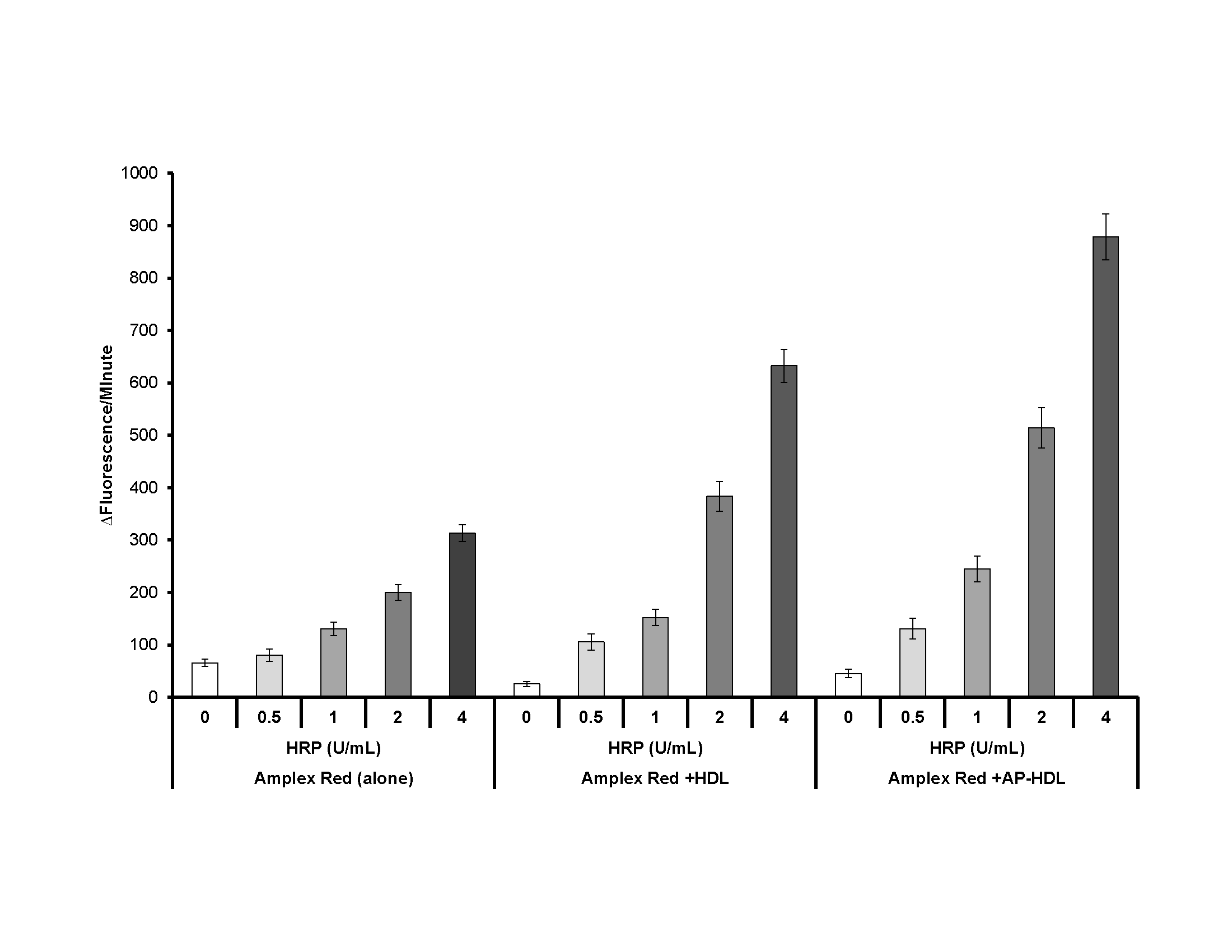

Supplement: Figure S2 — Without HRP lipid probe interactions are present but increasing amounts of HRP can increase the efficiency of detection of hydroperoxides carried by a specific amount of HDL cholesterol. In a 96 well flat bottom plate, 50 ul of 1X reaction buffer was added to each well alone or with 5 µg (cholesterol) of apoB depleted serum (as determined by a cholesterol assay) from a donor with anti-inflammatory HDL (HDL) and from a donor with acute phase HDL (AP-HDL), each in quadruplicates. 50 µl of HRP (0.5–4 U/ml) was then added to all wells followed by incubation at 37°C for 60 min. 50 µl of Amplex Red Reagent (final concentration 300 µM) was then added to each well for a total volume of 150 µl and the rate of production of resorufin was followed at 37°C in one-minute intervals for 60 minutes. The rates of change in fluorescence between 0 and 60 minutes are plotted for the quadruplicates, as well as means/standard deviations. In the absence of HRP, fluorescence quenching and lipid-probe interactions lead to reduction in the fluorescence readout after addition of a specific amount of HDL cholesterol compared to the fluorochrome alone, consistent with our previous observations with other fluorochromes such as DHR and DCF. Addition of ≥2 U/ml of HRP lead to a specific amplification of the quantification of the hydroperoxides associated with a specific amount of HDL cholesterol. A representative sample from each type of HDL (HDL vs AP-HDL) is shown and similar results were observed for 5 different other samples (5 HDL and 5 AP-HDL). In addition similar results with HRP were observed when 5 µg of HDL cholesterol isolated by FPLC or ultracentrifugation were added to the reaction. (TIFF) [file pone.0111716.s002.tiff]

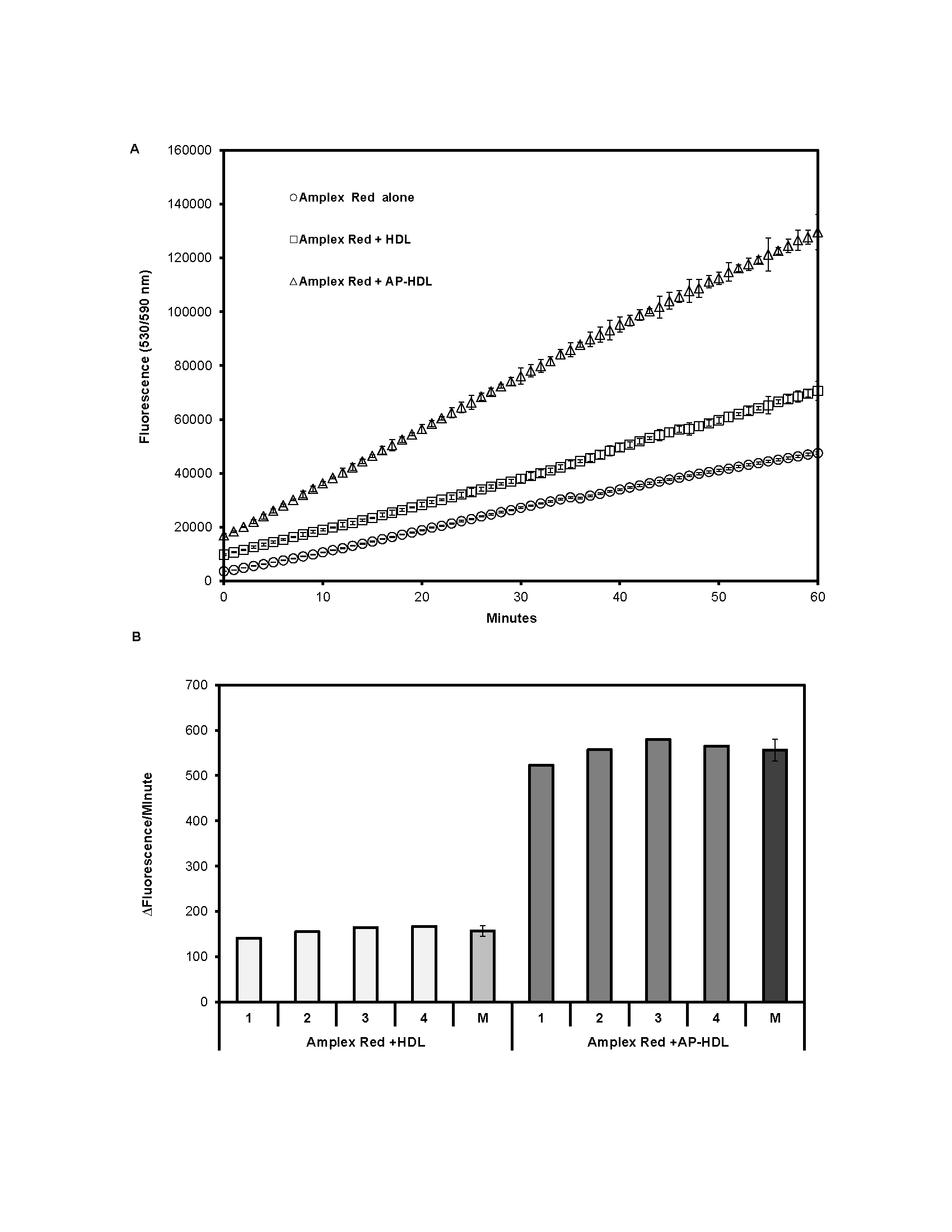

Supplement: Figure S3 — Oxidation of Amplex Red and effect of added HDL. In a 96 well flat bottom, 50 µl of 1X reaction buffer (0.5 M potassium phosphate, pH 7.4, 0.25 M NaCl, 25 mM cholic acid, 0.5% Triton X-100) was added to each well alone or with 5 µg (cholesterol) of apoB depleted serum (as determined by a cholesterol assay) from a donor with anti-inflammatory HDL (HDL) and from a donor with acute phase HDL (AP-HDL), each in quadruplicates. 50 µl of HRP was then added to all wells followed by incubation at 37°C for 60 min. 50 µl of Amplex Red Reagent (final concentration 300 µM) was then added to each well for a total volume of 150 µl. The rate of production of resorufin was followed at 37°C in one-minute intervals using a fluorescence microplate reader set to detect 530/590 nm excitation/emission. A. The means and standard deviations of the quadruplicate fluorescence measurements are plotted over time. B. The rates of change in fluorescence between 0 and 60 minutes (calculated by linear regression) are plotted for the quadruplicates, as well as means/standard deviations. The background fluorescence of the blank well (no HDL) was subtracted from the readout of each well for each timepoint. (TIF) [file pone.0111716.s003.tif]

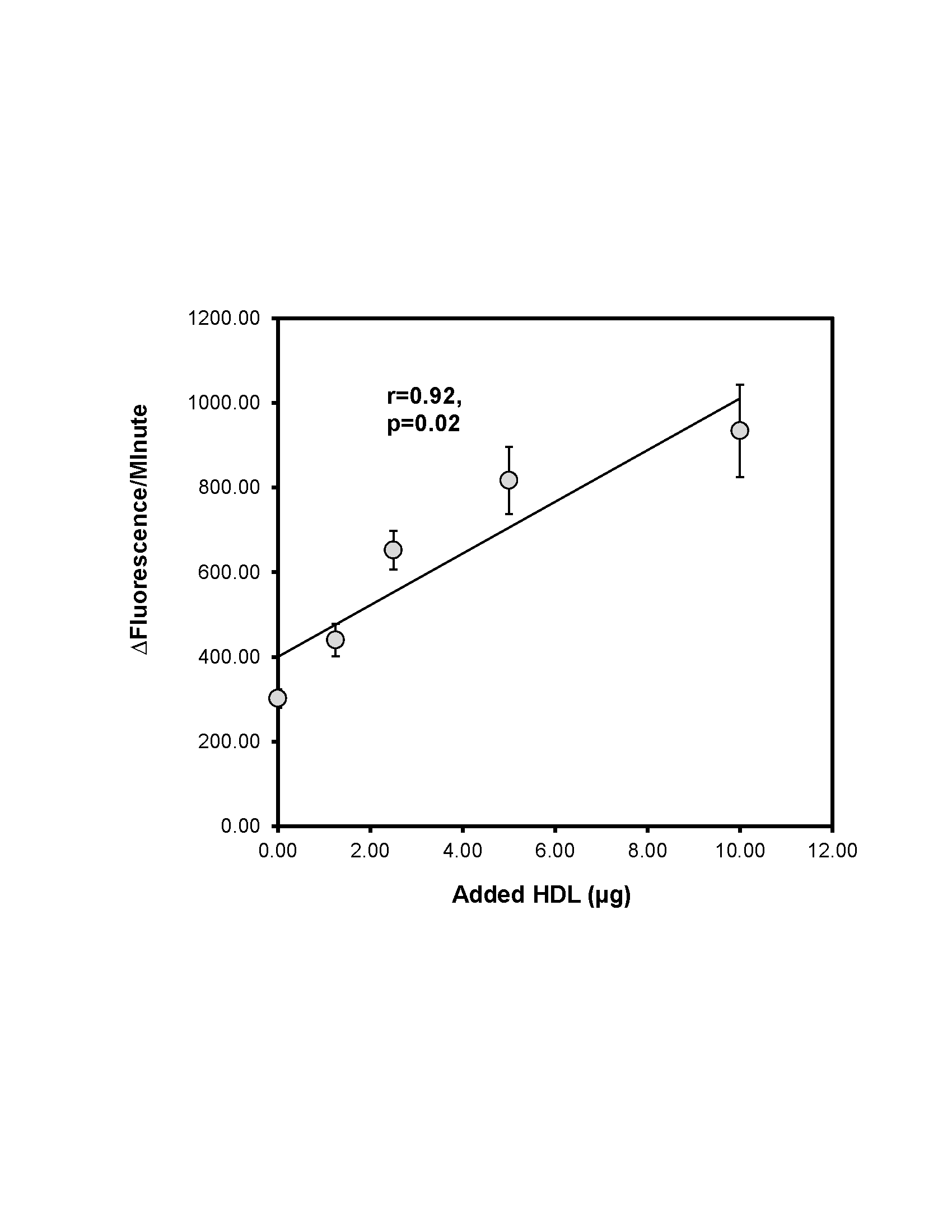

Supplement: Figure S4 — The Amplex Red assay can detect a concentration dependent increase in the amount of hydroperoxides associated with increasing amount of added HDL cholesterol. HDL isolated by ultracentrifugation was added in varying concentrations (cholesterol) to 300 µM Amplex Red in a 96 well flat bottom plate and the rate of change in fluorescence was measured as in Fig. 2 in the presence of 4 U/ml of HRP. The rates of change in fluorescence (means and standard deviations) are plotted against the amounts of added HDL. In addition similar results with HRP were observed when HDL cholesterol isolated by PEG precipitation was added to the reaction. There was a concentration dependent increase in the fluorescent readout with increasing amount of added HDL cholesterol in the presence of HRP in contrast to a concentration dependent decrease in the readout with increasing amount of added HDL cholesterol with other fluorescent probes (DCF and DHR). (TIFF) [file pone.0111716.s004.tiff]

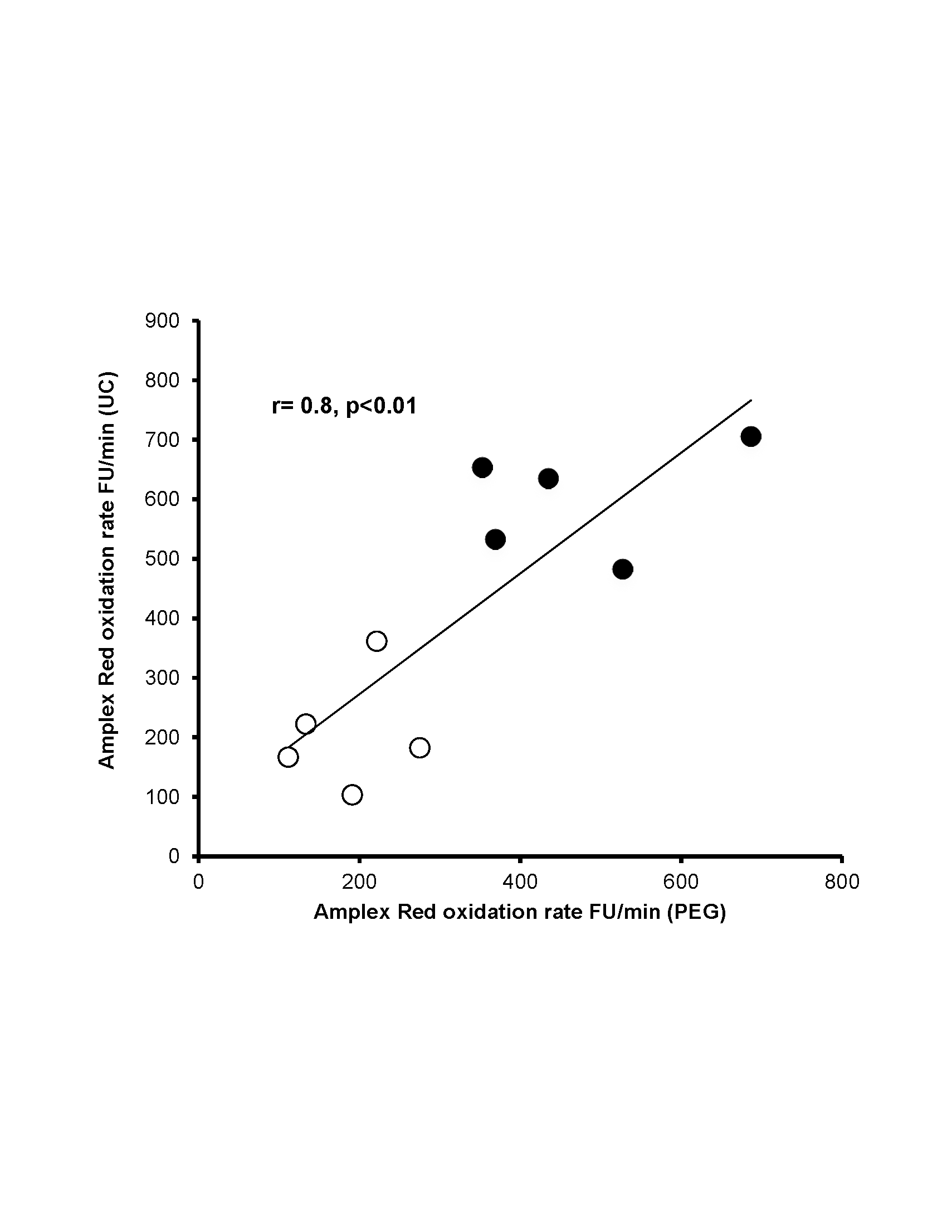

Supplement: Figure S6 — Correlation of effect of HDL on Amplex Red oxidation using different methods of HDL isolation. HDL was isolated by ultracentrifugation or PEG precipitation from 5 HIV infected patients known to have acute phase HDL (AP-HDL; shown in solid black circles) and 5 patients with normal HDL (shown in white circles). 5 ug of HDL cholesterol was then added to 300 µM Amplex Red in a 96 well flat bottom plate and the rate of change in fluorescence was measured as in Fig. 2 in the presence of 4 U/ml of HRP. The mean rates of change in fluorescence are plotted. (TIFF) [file pone.0111716.s006.tiff]

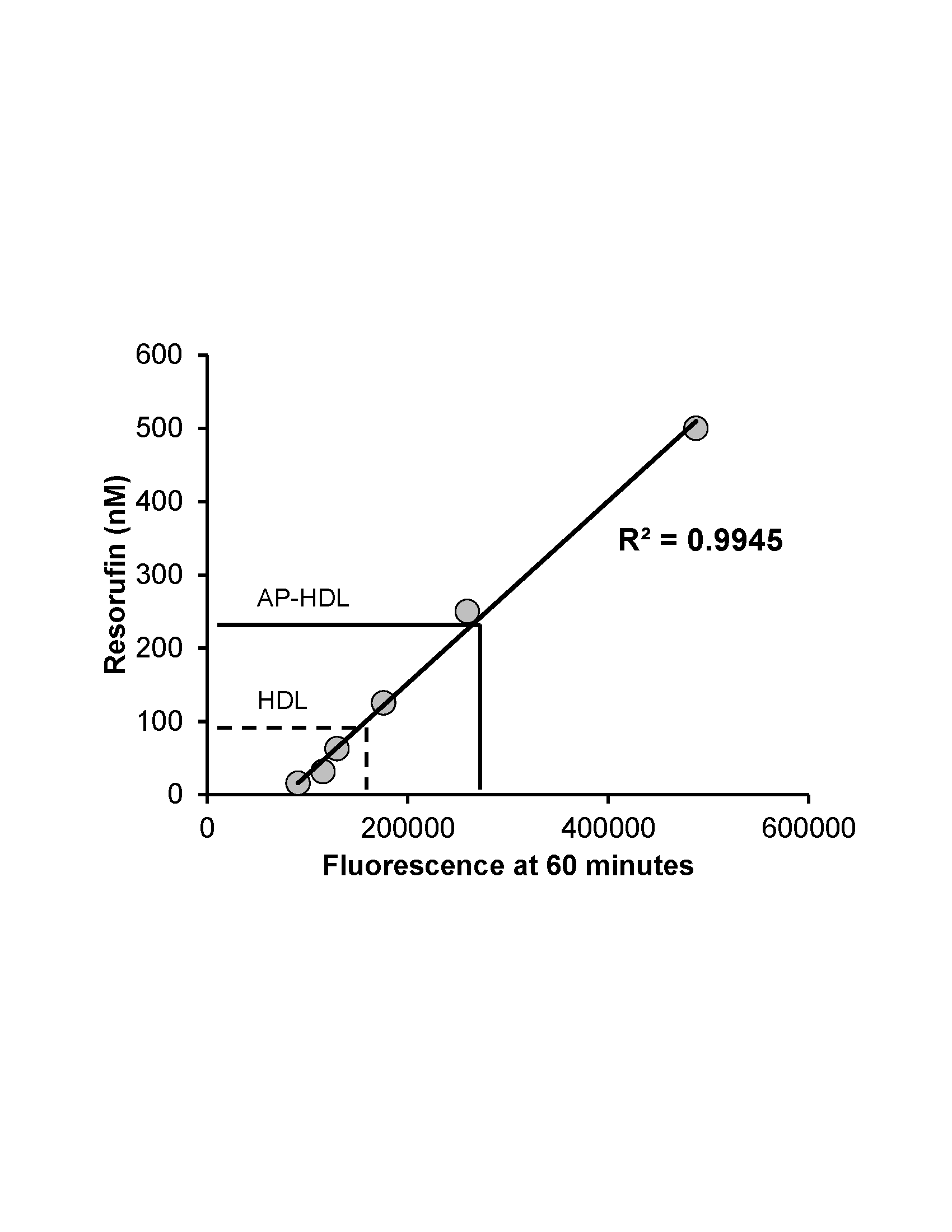

Supplement: Figure S7 — Commercially available resorufin standards can be used to standardize fluorescence-based quantification of the hydroperoxide content of a specific amount of HDL cholesterol. A commercially available resorufin fluorescence reference standard can be used to prepare a standard curve to determine the moles of fluorescent product produced in the Amplex Red reaction according to the manufacturer's instructions. Endpoint measurement of the fluorescence signal that corresponds to production of resorufin and oxidation of the Amplex Red reagent was performed at 60 minutes as described in Fig. 2. The reference 2 mM resorufin standard was diluted accordingly to generate a standard curve of resorufin that would “fit” the dynamic range of the measured fluorescence at 60 minutes for the specific assay. Towards this end, the amount of the added cholesterol and the time of the reaction for certain photomultiplier sensitivity needs to be titrated carefully. The triplicate fluorescence readings for each standard were averaged and the mean fluorescence was calculated. The average fluorescence of the blank sample (Amplex Red alone without HDL) was subtracted from all the standards and samples and the adjusted fluorescence was calculated. The adjusted fluorescence of the standards was plotted as a function of the concentration of the resorufin standards. An example of a standard curve with a dynamic range 15.625–500 nM and six standards is shown. The fluorescence of the HDL samples was calculated in the presence of 5 µg (cholesterol) of added HDL. HDL was isolated by PEG precipitation from HIV infected subjects with acute phase HDL (AP-HDL) and healthy subjects with normal HDL. The means of quadruplicates were calculated (adjusted fluorescence). The amount of produced resorufin for each HDL sample was calculated using the equation obtained from the linear regression of the standard curve substituting adjusted fluorescence values for each sample. 2 representative samples (one with normal [file pone.0111716.s007.tiff]

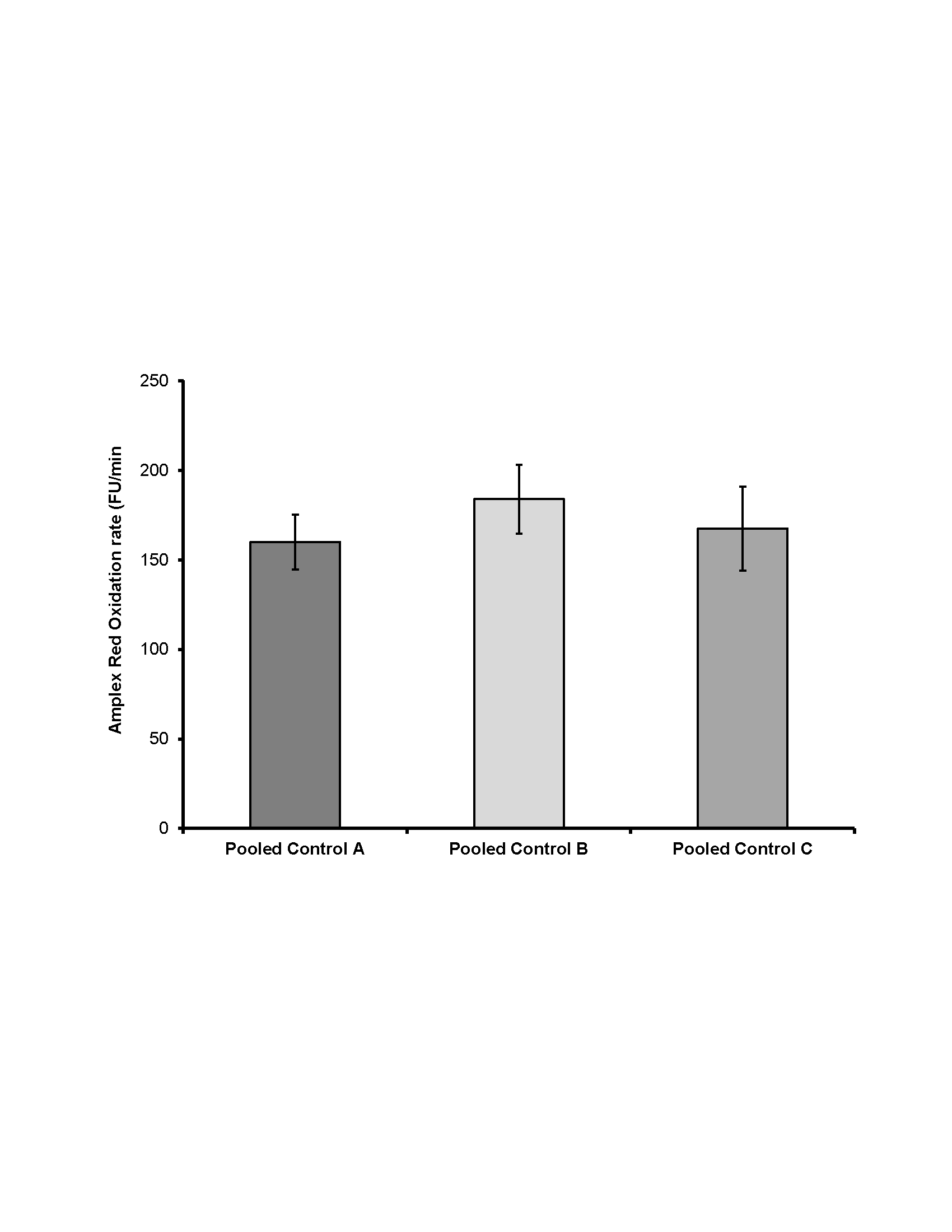

Supplement: Figure S9 — A specific amount of HDL cholesterol isolated from pooled blood bank specimens of healthy subjects can be used as a universal control to standardize the Amplex Red assay of HDL function. HDL was isolated using PEG precipitation from 3 different groups (A, B, C; each 30 samples) of cryopreserved serum blood bank specimens. The HDL samples in each group were pooled as described in Fig. S6 (three different blood bank pools). The Amplex Red oxidation rate (AROR) was determined as described in Materials and Methods. The mean AROR among the 3 different blood bank pools was comparable. Thus, this current approach may be used to create a universal control for determination of DOR by combining HDL samples from at least 30 different donors. (TIFF) [file pone.0111716.s009.tiff]

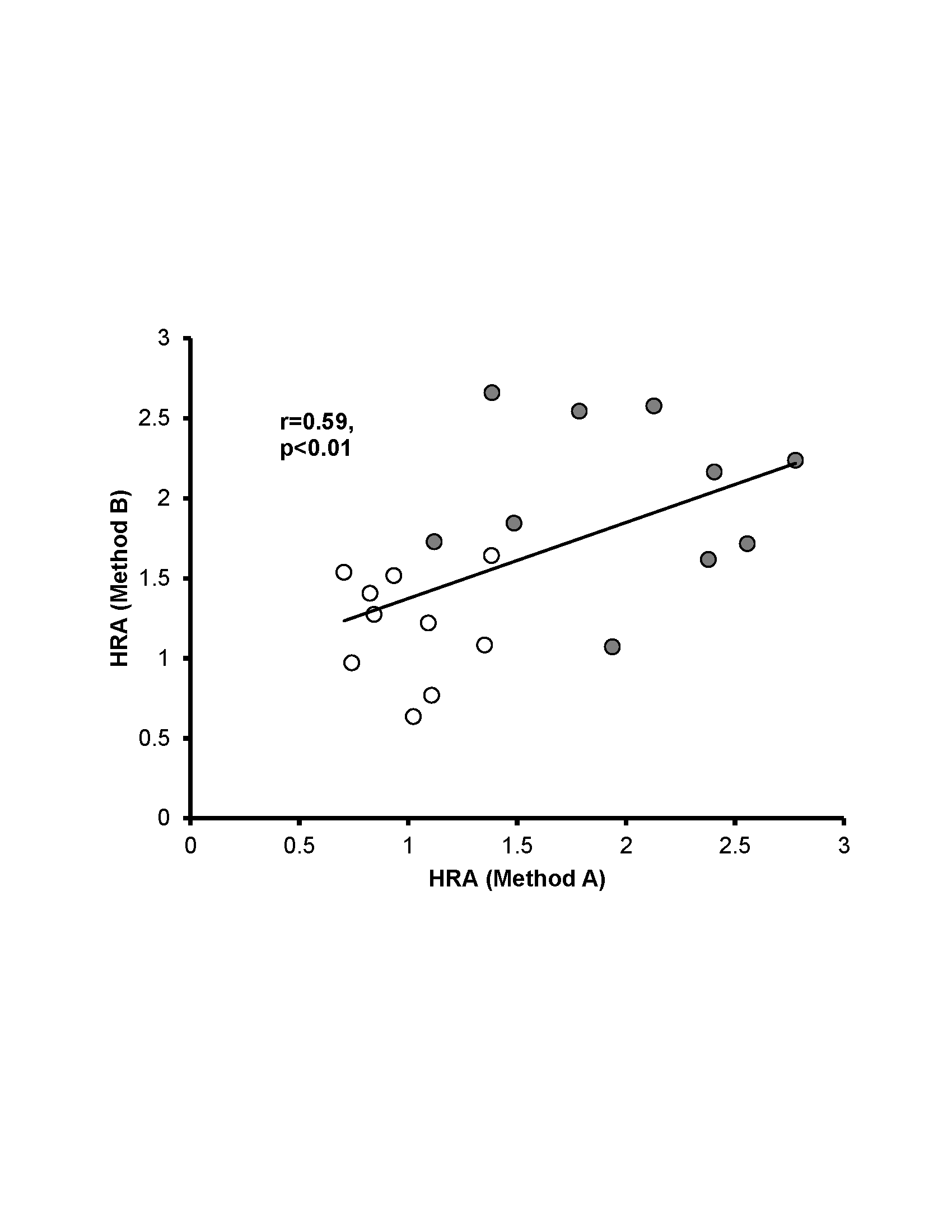

Supplement: Figure S10 — The HDL concentration as determined by the clinical laboratory can be used to adjust the fluorescence readout for the amount of HDL cholesterol in each sample in the Amplex Red assay of HDL function. ApoB depleted serum was isolated by PEG precipitation from 20 subjects (10 healthy and 10 with HIV infection and acute phase HDL). The Amplex Red oxidation rate (AROR) was determined as described in Fig. 2 and HDL was added using two different methods (A and B). In method A the HDL cholesterol concentration of each sample was determined using a cholesterol assay as described in the Methods section and then 5 µg of HDL cholesterol was added to each well. The individual normalized AROR (nAROR) [nAROR = (AROR/AROR control] is a measure of the HDL redox activity (HRA) and is evaluated as a ratio to the AROR of a control HDL isolated from pooled serum as described in Fig. S7. In Method B the HDL cholesterol concentration of each sample (mg/dl) was measured by the clinical lab and this value is routinely available in the setting of standard clinical care. A specific volume of apoB depleted serum (50 µl) was added to each well, the AROR for each sample was determined as above and this readout was normalized by the HDL cholesterol concentration of each sample (nHDLAROR). A control HDL sample was created after pooling equal volumes of apoB depleted serum from 30 healthy blood bank serum. The HDL concentration of this pooled HDL control was calculated from the HDL concentrations of the individual samples (measured in mg/dl by the clinical lab) and the fluorescence readout was normalized by this value (nHDLAROR control). The individual normalized to control AROR is evaluated as a ratio to the AROR of a control HDL isolated from pooled serum [nAROR = (nHDLAROR/nHDLAROR control]. The values represent means of triplicate samples and the correlation coefficient is shown. Data from healthy subjects are shown as white circles and data from HIV infected subjects are shown as gray circles [file pone.0111716.s010.tiff]

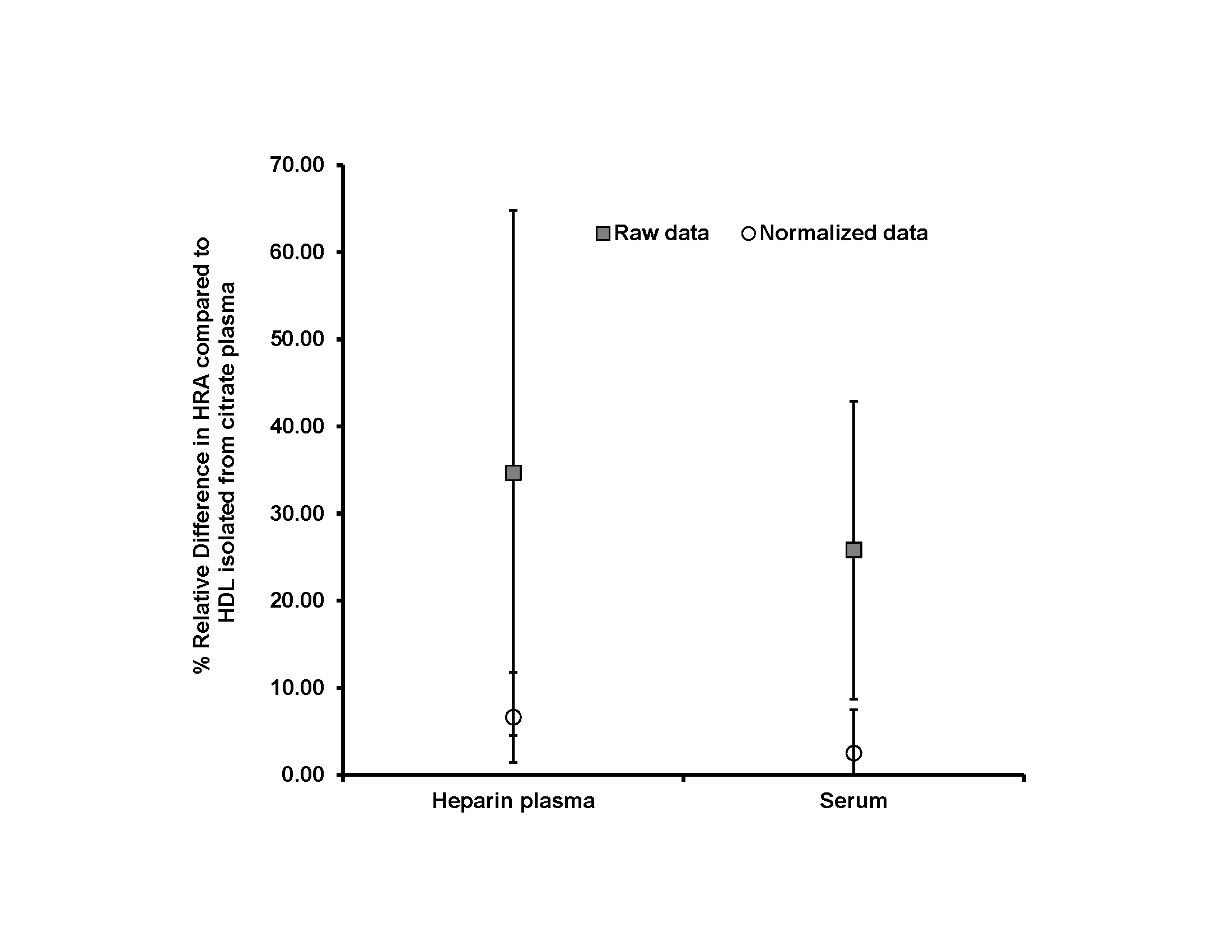

Supplement: Figure S12 — The standardization method minimizes the effect of different matrices on oxidative properties of HDL. Oxidation rate of Amplex Red (AROR) in the presence of 13 different samples of HDL [7 from patients with Human Immunodeficiency virus (HIV-1) infection and 6 from healthy volunteers (Non HIV)] isolated by PEG precipitation from heparin plasma, citrate plasma and serum was assessed as described in Methods and in Fig. 2. The values represent means of all the samples. The HRA values from plasma citrate samples correlated significantly (p<0.01) with the HRA values from serum samples but heparin interfered with the readout. In addition, the HRA of each sample was normalized by the HRA value of the control sample and the % relative HRA was determined as in Fig. S10. The suggested standardization method improved the correlations of the HRA values and tended to minimize the effect of different matrices on determination of HRA. (TIFF) [file pone.0111716.s012.tiff]

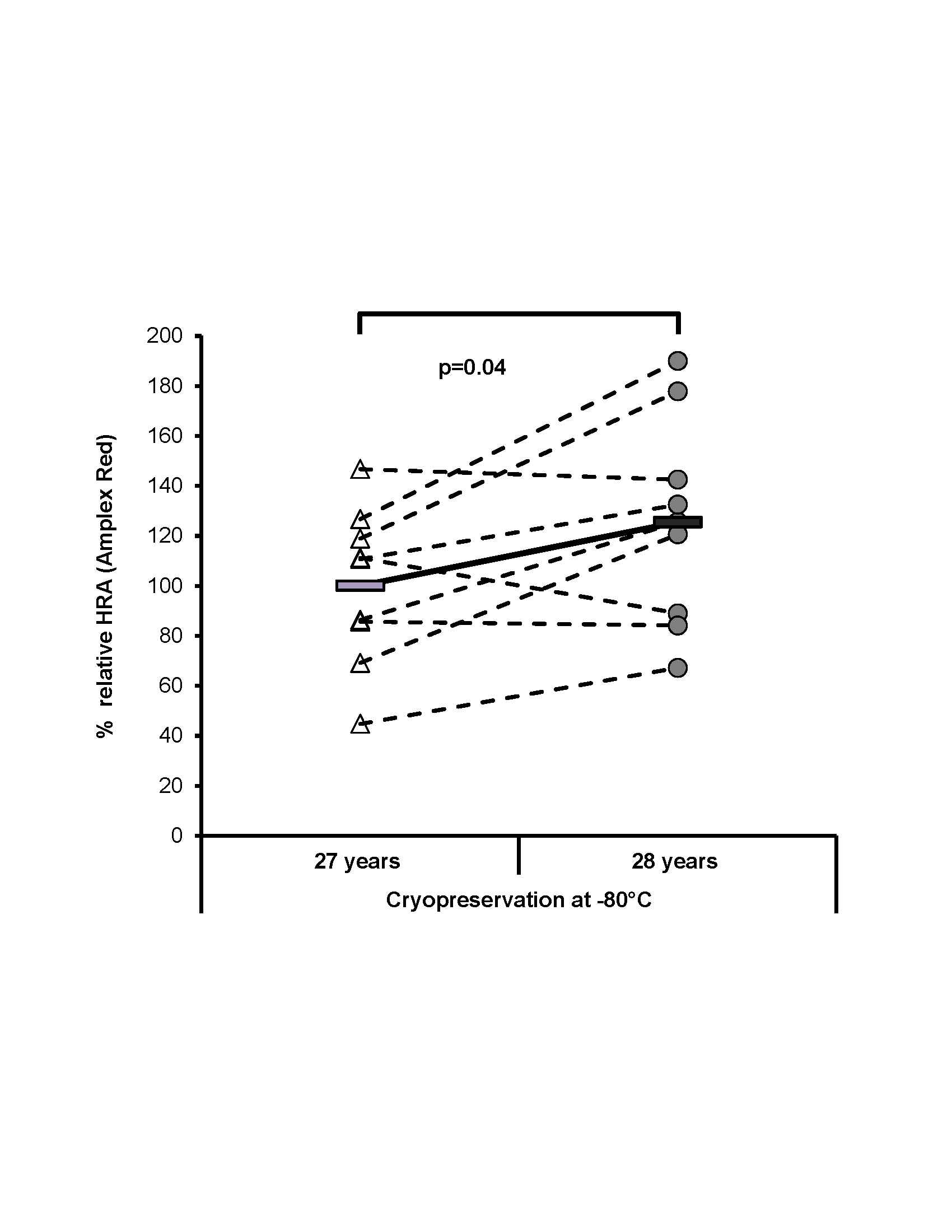

Supplement: Figure S13 — Long term storage of blood specimens tends to increase HDL redox activity (HRA) as determined by the Amplex Red assay but the results are comparable between different timepoints. The Multicenter AIDS Cohort Study (MACS) has defined a group of men who remained HIV-1- seronegative despite hundreds to thousands of high-risk sexual exposures in the 1980s. The MACS cohort recruited men in 1985 for natural history studies (Am J Epidemiol 126: 310-8), and has continued to follow subjects every 6 months to the present. Using 9 stored serum samples from this cohort that were stored for 27 and 28 years at −80°C, we determined the effect of long term storage at −80C on HDL redox activity (HRA) as described in Fig. 2. The readout of each sample was expressed as % relative to the average readout of all 9 samples at 27 years of cryopreservation. The HRA as determined by the Amplex Red assay significantly increased after cryopreservation for one extra year (125±41% vs 100±31%, p value for paired t test = 0.04) and the readouts from the 2 groups correlated significantly (r = 0.69, p<0.01). (TIFF) [file pone.0111716.s013.tiff]

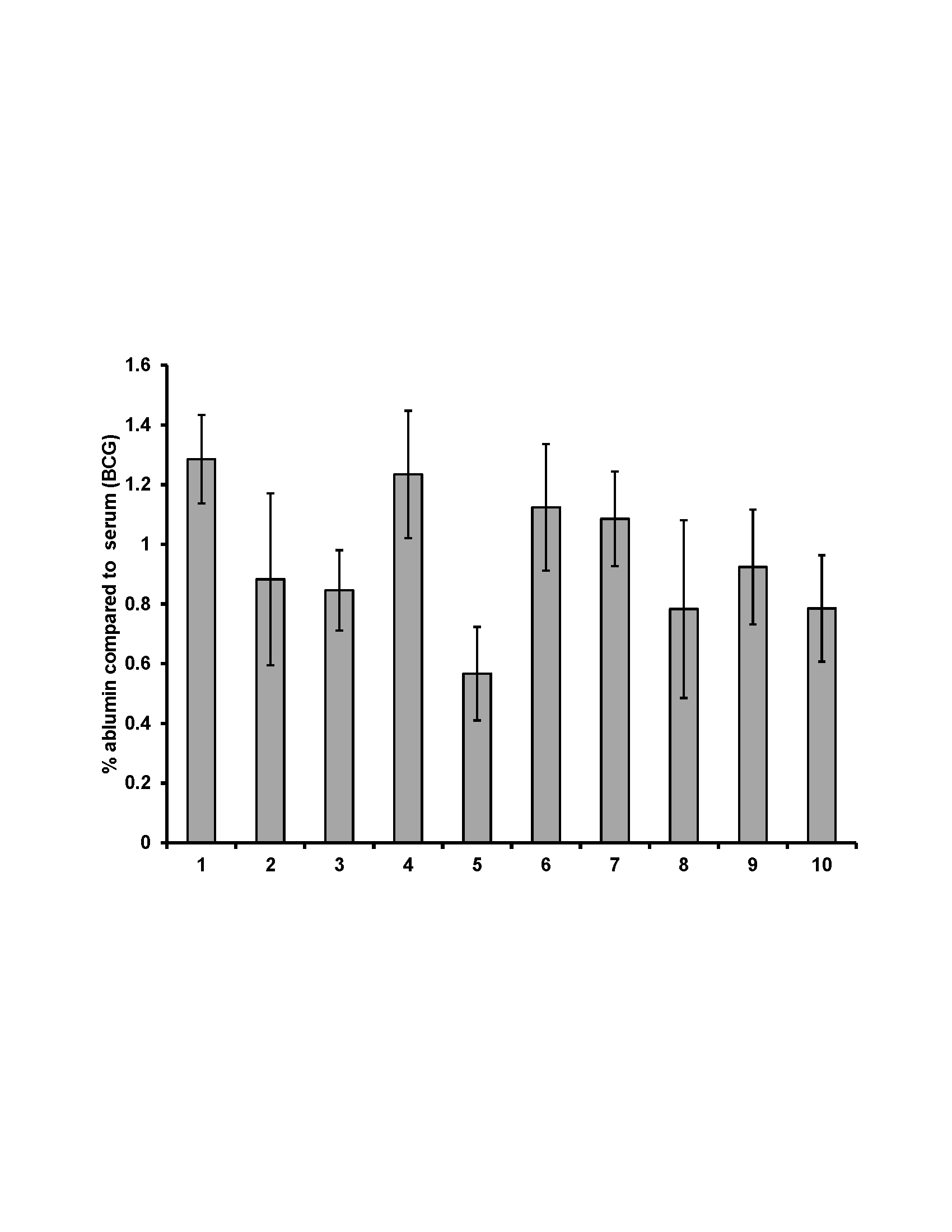

Supplement: Figure S14 — HDL isolated using immunoaffinity capture of HDL is largely free of albumin. 50 ul of plasma (n = 10) was added into 96 wells and was isolated using immunoaffinity capture of HDL according to the manufacturer's instructions (Kit A). After 5 washes, 300 µl of albumin bromocresol green reaction (BCG) reagent (Thermo Scientific Inc) were added to each well and after 90 second incubation at 37°C the optical density at 630 nm was read. Results are expressed as % relative value compared to the positive control (50 µl of plasma). The median relative albumin content bound to HDL was 0.90% with the BCG method, one of the most sensitive and specific methods to detect albumin. The minimal detection of HDL-bound albumin (<0.5% relative to the positive control) was also confirmed with a secondary antibody against albumin conjugated to horseradish peroxidase (HRP) (Pierce Inc). Similar results were obtained with Kit B. (TIFF) [file pone.0111716.s014.tiff]
