# Supplementary material for: A High Throughput Biochemical Fluorometric Method for Measuring Lipid Peroxidation in HDL
Source: PLoS One. 2014 Nov 4;9(11):e111716. doi: 10.1371/journal.pone.0111716 (PMC4219769; doi:10.1371/journal.pone.0111716)
Supplement: Table S1 — Outline of protocol used to measure HDL lipid peroxidation (PEG method for HDL isolation). (DOCX) [file pone.0111716.s015.docx]

**Table S1**

|  | **Outline of protocol used to measure HDL lipid peroxidation (PEG method for HDL isolation)** |
| --- | --- |
| **Preparation of apoB depleted serum** | Isolate apoB depleted serum by mixing volume of plasma/serum and PEG 1:1 e.g. 100 ul of plasma plus 100 ul PEG. Centrifuge at 1000-2000 g for 10 minutes to prepare apoB depleted serum. |
| **Preparation of HRP/catalase solution** | Prepare HRP and catalase solutions of >1 U/ml (titrate amount so the fluorescence signal of the samples is above the blank well) in saline buffer. Note that the HRP and catalase will be used before the amplex Red reagent and should not be added in the amplex red reagent. |
| **Preparation of resorfurin standards (optional)** | A 2 mM resorufin solution was used to prepare a standard curve to determine the moles of product produced in the Amplex® Red reaction. The appropriate amount of 2 mM resorufin reference standard should be diluted into 1X reaction buffer to produce resorufin concentrations of 0 to ~20 μM). |
| **Preparation of positive (optional) and negative controls** | 1X reaction buffer without cholesterol was used as a negative control. A 20 mM H2O2 working solution can used as a positive control. |
| **Addition of samples** | Add 50 ul of the supernatant (apoB depleted serum), to 96-well plates (polypropylene, flat bottom, black, Fisher Scientific, USA) in quadruplicates. |
| **Addition of HRP** | Add 50 ul of HRP to all wells and incubate for 60 minutes at 37 ◦C. Do not discard samples after incubation (no wash steps between addition of reagents) |
| **Addition of Amplex Red** | Add 50 ul of 300 μM of Amplex Red reagent (Invitrogen) to each microplate well containing the samples and controls. |
| **Measurement of fluorescence** | Assess the fluorescence of each well at one-minute intervals over 60 minutes with a plate reader (Biotek, Vermont, USA), using a 530/590 nm filter pair. |
